# Permissive selection followed by affinity-based proliferation of GC light zone B cells dictates cell fate and ensures clonal breadth

**DOI:** 10.1073/pnas.2016425118

**Published:** 2021-01-08

**Authors:** Rinako Nakagawa, Amparo Toboso-Navasa, Marta Schips, George Young, Leena Bhaw-Rosun, Miriam Llorian-Sopena, Probir Chakravarty, Abdul Karim Sesay, George Kassiotis, Michael Meyer-Hermann, Dinis Pedro Calado

**Affiliations:** ^a^Immunity and Cancer Laboratory, Francis Crick Institute, London NW1 1AT, United Kingdom;; ^b^Department of Systems Immunology and Braunschweig Integrated Centre for Systems Biology, Helmholtz Centre for Infection Research, 38106 Braunschweig, Germany;; ^c^Retroviral Immunology Laboratory, Francis Crick Institute, London NW1 1AT, United Kingdom;; ^d^Advanced Sequencing Laboratory, Francis Crick Institute, London NW1 1AT, United Kingdom;; ^e^Bioinformatics and Biostatistics Laboratory, Francis Crick Institute, London NW1 1AT, United Kingdom;; ^f^Institute for Biochemistry, Biotechnology and Bioinformatics, Technische Universität Braunschweig, 38106 Braunschweig, Germany;; ^g^Peter Gorer Department of Immunobiology, School of Immunology & Microbial Sciences, King’s College London, London WC2R 2LS, United Kingdom

**Keywords:** GC B cells, positive selection, memory B cells, clonal diversity, affinity maturation

## Abstract

Affinity maturation depends on how efficiently germinal centers (GCs) positively select B cells in the light zone (LZ). Positively selected GC B cells recirculate between LZs and dark zones (DZs) and ultimately differentiate into plasmablasts (PBs) and memory B cells (MBCs). Current understanding of the GC reaction presumes that cMyc-dependent positive selection of LZ B cells is a competitive affinity-dependent process; however, this cannot explain the production of GC-derived lower-affinity MBCs or retention of GC B cells with varied affinities. Here, by combining single-cell/bulk RNA sequencing and flow cytometry, we identified and characterized temporally and functionally distinct positively selected cMyc^+^ GC B cell subpopulations. cMyc^+^ LZ B cell subpopulations enriched with either higher- or lower-affinity cells diverged soon after permissive positive selection. The former subpopulation contained PB precursors, whereas the latter comprised less proliferative MBC precursors and future DZ entrants. The overall affinity of future DZ entrants was enhanced in the LZ through preferential proliferation of higher-affinity cells. Concurrently, lower-affinity cells were retained in GCs and protected from apoptosis. These findings redefine positive selection as a dynamic process generating three distinct B cell fates and elucidate how positive selection ensures clonal diversity for broad protection.

## Significance

Light zone (LZ) B cells are selected in germinal centers (GCs) in a cMyc-dependent manner, before dark zone (DZ) migration. Antigen affinity of B cells is enhanced during GC responses, and some differentiate into plasmablasts or memory B cells (MBCs). Currently, GC selection is presumed as a competitive affinity-dependent process. This cannot explain retention of GC B cells with varied affinities. We identified cMyc^+^ LZ B cell subpopulations enriched with either higher-affinity plasmablast precursors or lower-affinity MBC precursors and future DZ entrants, which diverged soon after permissive selection. Future DZ entrants’ affinity was enhanced through preferential proliferation of higher-affinity cells, and lower-affinity cells were retained in GCs. These findings elucidate mechanistically how GC selection ensures clonal diversity for broad protection.

B cells substantially increase their affinity for the antigen during the course of T cell-dependent immune responses. This fine-tuning of antibody specificity is known as affinity maturation (1, 2) and occurs in germinal centers (GCs) through a bidirectional migration cycle between two phenotypically and anatomically distinct regions of GCs, light zone (LZ) and dark zone (DZ) (3). GC B cells exit cell cycle upon their transit from the DZ to the LZ where antigen-driven selection occurs in response to signals from B cell receptor (BCR) engagement and T follicular helper cells (TFHs). Positively selected GC B cells in the LZ acquire cMyc (avian myelocytomatosis virus oncogene cellular homolog) expression, a critical regulator for cell proliferation and GC maintenance, as “licensed” cells (4, 5), which are protected from apoptosis (6). The transient cMyc expression leads the positively selected LZ B cells to reenter the DZ where cells proliferate and hypermutate their BCRs to improve affinity for the antigen.

In the currently favored model of affinity maturation, positive selection occurs in an affinity-dependent manner in the LZ (7, 8). GC B cells retrieve antigen displayed on follicular dendritic cells (FDCs), internalize the BCR that binds antigen, and present differing levels of antigen in the form of peptide major histocompatibility II in proportion to its affinity, which allows clones with relatively higher BCR affinity to receive more help from TFHs whose number is limited (8–10). Indeed, positively selected cMyc^+^ GC B cells in the LZ express BCRs with higher affinity for the antigen compared with cMyc-negative LZ cells (4). cMyc^+^ GC B cells in the LZ restart the cell cycle and are primed to enter the DZ to complete cell division (4, 5, 11). Hence, the competitive advantage of the selected higher-affinity BCR clones is thought to occur only in the DZ where they undergo more rounds of cell division in enhanced tempo during prolonged dwell time (12, 13). However, this model cannot explain recent findings showing that GCs can retain cells expressing low- and moderate-affinity BCRs while antibody affinity is improved (14, 15). Additionally, within this model the roles of dividing LZ B cells that were observed in histology and two-photon microscopy experiments (7, 16–19) remain unknown.

cMyc expression in GC B cells effectively defines positively selected B cells (4, 5). However, this population is suggested to be developmentally heterogeneous (20, 21), with at least one of the subfractions requiring strong signals derived from TFHs for its generation (21). Consistent with this, increased TFHs help drives plasmablast (PB)/plasma cell (PC) differentiation from GC B cells (10) that carry higher-affinity BCRs (22) through recently identified PC precursors (Bcl6^lo^CD69^hi^ LZ GC B cells) expressing cMyc transcripts at a relatively higher level than the other LZ B cells (23). Taken together, these observations suggest that the cMyc^+^ compartment contains PB/PC precursors as well as DZ entrants and therefore, is heterogeneous regarding B cell differentiation stage and BCR affinity. In contrast to PBs/PCs, memory B cells (MBCs) appear to be formed in response to a minimal amount of help from TFHs (24) and predominantly contain lower-affinity clones (24, 25). Together with the observation that MBC precursors are relatively quiescent (24–26), MBCs are generally thought to arise from “nonpositively selected” LZ B cells. However, some MBC precursors have been found to carry higher-affinity BCRs (25), suggesting that a fraction of MBCs may also arise from positively selected LZ B cells. However, it remains unclear how MBCs are formed and thus, whether MBC precursors can emerge from positively selected cells.

In this study, we identified positively selected cMyc^+^ LZ B cells that comprise subpopulations representing three distinct B cells fates (pre-PB/PCs, pre-MBCs, and DZ entrants), and these subpopulations emerged in a sequential order following cMyc-inducing signals. In-depth analyses of these subpopulations demonstrated that GC positive selection is permissive to retaining a large proportion of low-affinity clones that are protected from apoptosis despite their low-affinity BCR. Sequential molecular and mitotic events in the cMyc^+^ LZ B cell compartment determined B cell fates and improved affinity of future DZ entrants. Hence, the manner of such dynamic positive selection can be beneficial to maintain clonal breadth and also advantageous for selecting antigen-specific cells to differentiate into MBCs.

## Results

### Identification and Flow Cytometric Delineation of Positively Selected cMyc^+^ GC B Cell Subpopulations.

**Fig. 1.**
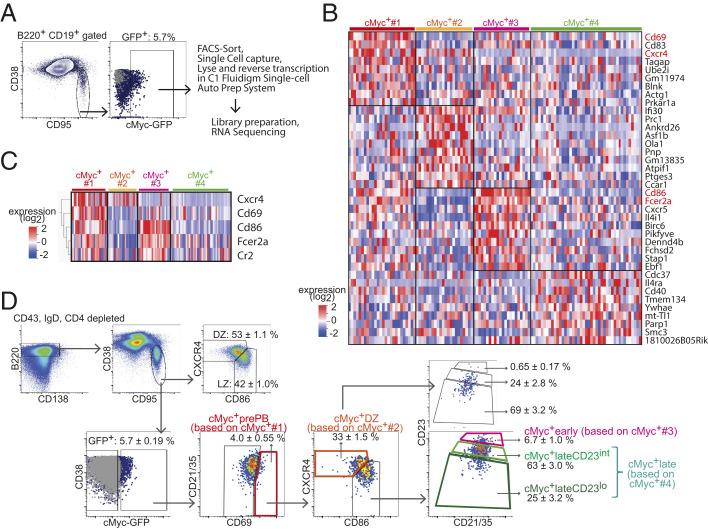
Identification and flow cytometric delineation of positively selected cMyc^+^ GC B cell subpopulations. (*A*) scRNAseq workflow and gating strategy for isolating splenic cMyc^+^ GC B cells of cMyc^gfp/gfp^ mice. (*B*) Heat map illustrating highly representative DEGs in each cluster. These DEGs passed thresholds: first, the cutoff for *P* ≤ 0.05 by multigroup comparison and second, for *P* ≤ 0.05 by two-group comparison (the cluster vs. ≠ the cluster) and log_2_ fold change >1. *Fcer2a* was the eighth enriched gene in the cMyc^+^#1 cluster, but it is not listed in the cluster because it is shown as the second enriched gene in the cMyc^+^#3 cluster instead. Genes encoding key markers that are used for delineating flow cytometric cMyc^+^ GC B cell subpopulations (as described in *D*) are highlighted in red. (*C*) Heat map illustrating the five key marker genes used in *D*. The markers were selected from 76 DEGs (*P* ≤ 0.005, by multigroup comparison). (*D*) Representative flow cytometry plots illustrating gating strategy to delineate cMyc^+^ GC B cell subpopulations. Splenic GC B cells of C57BL/6 mice are shown in gray dots as cMyc-GFP^neg^ control cells. Average ± SEM value (15 mice) is shown in each indicated gate. Splenic GC B cell response to SRBC on day 7 in cMyc^gfp/gfp^ mice.

To investigate the heterogeneity of cMyc^+^ GC B cell compartment, we utilized cMyc^gfp/gfp^ reporter mice that express a GFP-cMyc fusion protein from the endogenous cMyc locus and allow monitoring of cMyc expression by GFP fluorescence (27). Splenic cMyc^+^ GC B cells from cMyc^gfp/gfp^ mice were sorted by flow cytometry 10 d after immunization with (4-hydroxy-3-nitrophenyl)acetyl (NP) hapten conjugated to chicken gamma globulin (NP-CGG) and were subjected to single-cell RNA sequencing (scRNAseq) (Fig. 1*A*). Transcriptomic profiling and unsupervised clustering of 205 cells revealed 678 differentially expressed genes (DEGs) by multigroup comparison (*P* ≤ 0.05) and five clusters (cMyc^+^#1, #2, #3, #4, and #5) within cMyc^+^ GC B cells (*SI Appendix*, Fig. S1 *A* and *B*). The t-Distributed Stochastic Neighbor Embedding (t-SNE) plot indicated that cells in the cMyc^+^#5 cluster represented an intermediate state between the cMyc^+^#4 and cMyc^+^#2 clusters (*SI Appendix*, Fig. S1*A*). We excluded the cMyc^+^#5 cluster from the scRNAseq dataset and focused on cMyc^+^ clusters #1 to #4 for further analysis (Fig. 1*B* and *SI Appendix*, Fig. S1*C*) because clear-cut markers could not be identified for the cMyc^+^#5 cluster (*SI Appendix*, Fig. S1*B*). Transcriptomic analysis of cells in these clusters revealed 547 DEGs by multigroup comparison (*P* ≤ 0.05), and 9 or 10 representative DEGs per cluster were depicted after further filtering out the DEGs by two-group comparison (*P* ≤ 0.05) with more than twofold change (Fig. 1*B*). The representative DEGs of the clusters included activation marker genes (*Cd86*, *Cd83*, and *CD69*), early-response genes (*Nfkbia*), genes involved in cell cycle regulation (*Ccnd2* and *CDC37*), immunoregulatory molecules (*Fcer2a*; encoding CD23 and *Cr2*; encoding CD21/CD35), and chemokine receptors (*Cxcr5* and *Cxcr4*) (Fig. 1*B*). It has been shown that *Cd86*, *Cd83*, *Fcer2a*, and *Cxcr5* are highly expressed in LZ B cells (10) and that *CD69*, *Nfkbia*, and *Ccnd2* are up-regulated in cMyc^+^ GC B cells (4, 5). To investigate distinctive biological features of the clusters, we performed Gene Set Enrichment Analysis (GSEA). This analysis revealed that the cMyc^+^#1 cluster appeared to be associated with PBs/PCs due to the high level of expression of the genes involved with interleukin (IL)-6 production and activation of nuclear factor kappa B (NF-κB) signaling, which are known to have critical roles for differentiation of PBs/PCs (28, 29) (*SI Appendix*, Fig. S1*B*). The cMyc^+^#1 cluster also expressed *Cd69* at the highest level among all the clusters (Fig. 1*B*), which was concordant with previously reported PB/PC precursors (23). In the cMyc^+^#2 cluster, genes associated with stress response caused by DNA damage and oxidation were significantly enriched (*SI Appendix*, Fig. S1*D*). Responses to DNA damage and oxidative stress are known to occur mainly in the DZ (30, 31), suggesting that the cMyc^+^#2 cluster represented cMyc^+^ DZ B cells. The cMyc^+^#3 cluster was characterized by high expression of the LZ markers *Fcer2a* (CD23) and *Cd86* (Fig. 1*B*). The expression of CD23 on B cells is known to be increased by CD40 ligation (32) and IL-4 stimulation (33), suggesting that cells in the cMyc^+^#3 cluster represent a cMyc^+^ LZ B cell population that has received T cell help more recently. In support of this, the cMyc^+^#3 cluster was also found to be enriched with genes associated with both “B cell receptor signaling pathway” and “immunological synapse” and thus, may represent an LZ subpopulation in the very early activation following BCR engagement and GC B/TFH interaction (*SI Appendix*, Fig. S1*D*). The cMyc^+^#4 cluster was characterized by relatively high expression of *Cdc37*, an Hsp90 cochaperone, and was enriched in genes associated with protein synthesis (Fig. 1*B* and *SI Appendix*, Fig. S1*D*). Therefore, this cluster may represent a population of cMyc^+^ LZ B cells that had been activated less recently than the cMyc^+^#3 cluster and were preparing for intensive cycling in the DZ, which is in agreement with decreased expression of genes associated with “immunological synapse” in the cluster (*SI Appendix*, Fig. S1*D*).

Having identified DEGs using cMyc^+^ GC B cell scRNAseq data, we sought out surface markers to subdivide the heterogeneous cMyc^+^ compartment by flow cytometry. Other than the well-established DZ/LZ distinguishing markers, CXCR4 and CD86, three other surface markers CD69 [a marker for PB/PC precursors (23)], CD23, and CD21/35 were selected from the 76 DEGs determined by multigroup comparison (*P* ≤ 0.005) (Fig. 1*C*). High expression of *Cd69* was representative of the cMyc^+^#1 cluster, and *Fcer2a* and *Cr2* expression effectively distinguished between the cMyc^+^#3, cMyc^+^#4, and cMyc^+^#2 clusters by high, intermediate, and low expression, respectively (Fig. 1*C*). Using the combination of these five surface markers, cMyc-GFP^+^ GC B cells isolated from cMyc^gfp/gfp^ mice 7 d after sheep red blood cells (SRBCs) immunization were initially subdivided into four subpopulations. Hence, a candidate cMyc^+^ PB precursor subpopulation was identified as CD69^hi^cMyc^+^ GC B cells (“cMyc^+^prePB”; based on the cMyc^+^#1 cluster); the cMyc^+^ DZ subpopulation (based on the cMyc^+^#2 cluster) was defined by the DZ gating (CXCR4^hi^CD86^lo^) among cMyc^+^ GC B cells after gating out cells in the cMyc^+^prePB subpopulation; the cMyc^+^early subpopulation (based on the cMyc^+^#3 cluster) was identified as CD23^hi^CD21/35^hi^ LZ B cells among cMyc^+^ GC B cells after gating out cells in the cMyc^+^prePB and cMyc^+^ DZ subpopulations; and the cMyc^+^late subpopulation (based on the cMyc^+^#4 cluster) was identified as CD23^+/lo^CD21/35^+/lo^ LZ B cells in the remaining cMyc^+^ LZ B cells (Fig. 1*D*). We noted that the cMyc^+^late subpopulation consisted of a mixed population containing a major CD23^+^CD21/35^+^ subpopulation and a small tail-like CD23^lo^CD21/35^lo^ subpopulation. To improve the quality of upcoming analysis, we further subdivided the population into CD23^+^CD21/35^+^ and CD23^lo^CD21/35^lo^ subpopulations, which were termed as cMyc^+^lateCD23^int^ and cMyc^+^lateCD23^lo^, respectively. In agreement with CD23 being highly expressed in LZ B cells (10), CD23 expression was down-regulated in the cMyc^+^ DZ subpopulation (Fig. 1*D*). Enumeration of the five cMyc^+^ GC B cell subpopulations showed that the cMyc^+^early and cMyc^+^prePB subpopulations were relatively rare within the cMyc^+^ compartment (451 ± 79.0 and 389 ± 60.7, respectively; 3 to 5% of the cMyc^+^ compartment). The largest population of the cMyc^+^ compartment was the cMyc^+^lateCD23^int^ subpopulation, followed by the cMyc^+^ DZ subpopulation (5,009 ± 871.9 and 3,691 ± 599.4; 37 to 53% and 28 to 39% of the cMyc^+^ compartment, respectively) (*SI Appendix*, Fig. S1*E*).

### cMyc^+^ LZ B Cell Subpopulations Emerge in a Sequential Order following Positive Selection Signals.

**Fig. 2.**
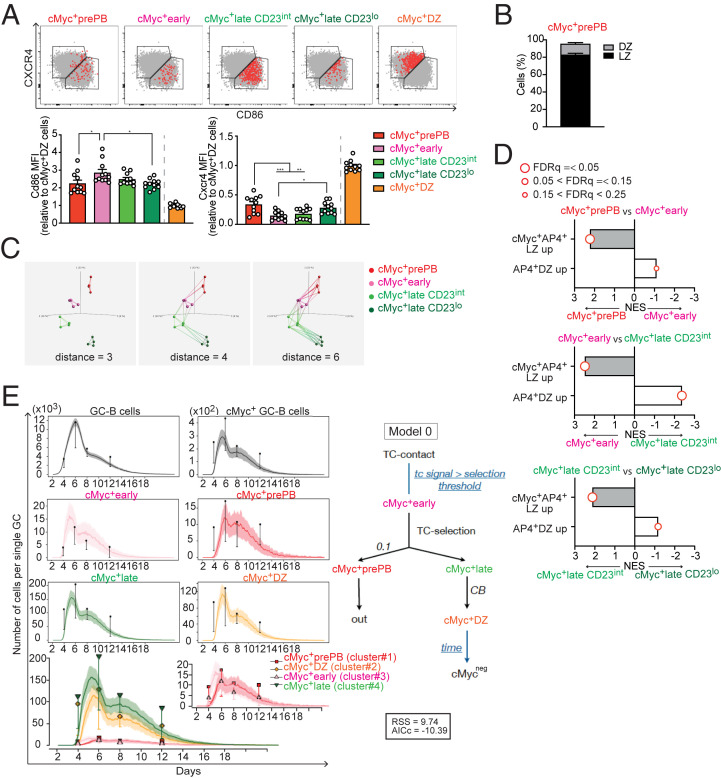
cMyc^+^ LZ B cell subpopulations emerge in a sequential order following positive selection signals. (*A*) Representative flow cytometry plots of CD86 vs. CXCR4 expression in the cMyc^+^ GC B cell subpopulations. Total GC B cells are shown in gray dots (*Upper*). Cd86 and Cxcr4 MFI values of the cMyc^+^ GC B cell subpopulations (relative to cMyc^+^ DZ values). (*B*) Percentages of cells in the cMyc^+^prePB subpopulation in DZ or LZ gates as described in *A*. Splenic GC B cell response to SRBC on day 7 in cMyc^gfp/gfp^ mice (*A* and *B*). Pooled data from more than three experiments with 11 (*A*) or 16 mice (*B*). Error bars indicate SEM. Statistics were calculated with one-way ANOVA. *P < 0.05; **P < 0.01; ***P < 0.001. (*C*) The 3D PCA plots of RNAseq data from the four cMyc^+^ LZ B cell subpopulations: cMyc^+^early, cMyc^+^lateCD23^int^, cMyc^+^lateCD23^lo^, and cMyc^+^prePB. The sample clusters were visualized by connecting each sample with its nearest neighbors for distance 3, 4, and 6. (*D*) Bar graphs representing GSEA of a cMyc^+^ LZ B cell subpopulation compared with the other (see description) for gene sets: “cMyc^+^AP4^+^ LZ up-regulated compared with AP4^+^ DZ” and “AP4^+^ DZ up-regulated compared with cMyc^+^AP4^+^ LZ.” The size of the dots indicates false discovery rate (FDR) q values. NES, normalized enrichment score. (*E*) In silico kinetics of the GC response (model 0) are plotted over the in vivo results for the number of GC B cells, cMyc^+^ GC B cells, and cells in each cMyc^+^ GC B cell subpopulation. cMyc positivity was attributed to the cells that were positively selected and received T cell signal (“tc signal”) from TFH above the selection threshold (= cMyc^+^early subpopulation). Cells in the cMyc^+^early subpopulation progressed to the cMyc^+^prePB or cMyc^+^late subpopulations, according to an in silico probabilistic fate decision between differentiation into GC output or CBs, respectively. Cells in the cMyc^+^late subpopulation progressed to the cMyc^+^ DZ subpopulation at the time of in silico CB differentiation. Cells in the cMyc^+^ DZ subpopulation lost cMyc expression after a fixed time. At the bottom plot, the four cMyc^+^ GC B cell subpopulations are plotted together, and *Inset* shows a magnification of cMyc^+^prePB and cMyc^+^early subpopulations. (*Right*) Working model of the cMyc^+^ GC B cell subpopulation dynamics in silico; RSS and AICc values are shown (see *SI Appendix*, Fig. S2 *B*–*E* for the other tested models). Free parameters used to fit the model are shown in blue letters. All data points were normalized with respect to the maximum value obtained in the simulation of the GC B cell kinetics in GC B cell numbers shown in the plot GC B cells. Mean (full lines) and SD (shaded area) of 100 simulations are shown. Black dots and colored dots represent in vivo data.

Having delineated cMyc^+^ GC B cell subpopulations by flow cytometry, we explored what these subpopulations represent. We hypothesized that these cMyc^+^ LZ B cell subpopulations represent different stages of the transition of GC B cells from the beginning of positive selection to DZ entry. To investigate the hypothesis, we examined the expression levels of DZ/LZ distinguishing markers CD86 and CXCR4 of cells in cMyc^+^ GC B cell subpopulations. We found that CD86 expression showed a downward trend in the following order: cMyc^+^early, cMyc^+^lateCD23^int^, and cMyc^+^lateCD23^lo^ (Fig. 2*A*). On the other hand, the CXCR4 expression was increasing in the same order (Fig. 2*A*). Accordingly, cells in the cMyc^+^lateCD23^lo^ subpopulation were found in the immediate vicinity of the border between the DZ and LZ (Fig. 2*A*); it appeared that these cells were about to enter the DZ. Although the DZ/LZ markers were not used for its delineation, the cMyc^+^prePB subpopulation was predominantly LZ B cells (82.9 ± 1.6%) (Fig. 2*B*), and most cells were clustered around the border between the DZ and LZ (Fig. 2*A*).

Next, we performed RNA sequencing (RNAseq) on the flow cytometry-delineated cMyc^+^ LZ B cell subpopulations and assessed the hypothesis by transcriptome analysis. To visualize how closely the subpopulations were correlated to each other, three-dimensional (3D) principle component analysis (PCA) plots were generated based on the distances in the space of all active variables (*P* ≤ 0.0005, multigroup comparison). In these PCA plots, each sample was connected with its nearest neighbors within the indicated distance. The analysis showed that each sample was tightly clustered within its own subpopulation (distance 3) (Fig. 2*C*). At a greater distance, the cMyc^+^early subpopulation was related to both the cMyc^+^prePB and cMyc^+^lateCD23^int^ subpopulations, and the cMyc^+^lateCD23^int^ subpopulation was connected with the cMyc^+^early and cMyc^+^lateCD23^lo^ subpopulations (distances 4 and 6) (Fig. 2*C*). We also carried out GSEA using gene signatures specific for subsets of GC B cells, cMyc^+^AP4^+^ LZ B cells, and AP4^+^ DZ B cells (34), which represent positively selected LZ B cells and DZ B cells emerging immediately after the transit of positively selected LZ B cells into the DZ, respectively. GSEA results between the subpopulations found to be closely related to each other in the PCA results (Fig. 2*C*) revealed that the cMyc^+^early subpopulation was significantly enriched for the cMyc^+^AP4^+^LZ GC B cell gene signature compared with cMyc^+^lateCD23^int^ and cMyc^+^lateCD23^lo^ subpopulations (Fig. 2*D*). The enrichment for this gene signature decreased in the cMyc^+^lateCD23^int^ subpopulation and more so in the cMyc^+^lateCD23^lo^ subpopulation (NES for cMyc^+^early vs. cMyc^+^lateCD23^int^ and cMyc^+^lateCD23^int^ vs. cMyc^+^lateCD23^lo^; 2.48 and 2.12, respectively) (Fig. 2*D* and *SI Appendix*, Fig. S2*A*). The opposite was true for the gene signature of AP4^+^ DZ GC B cells, which was significantly more enriched in the cMyc^+^lateCD23^lo^ subpopulation compared with the cMyc^+^lateCD23^int^ and cMyc^+^early subpopulations (Fig. 2*D* and *SI Appendix*, Fig. S2*A*). Notably, the enrichment for this gene signature decreased in the cMyc^+^lateCD23^int^ subpopulation and more so in the cMyc^+^early subpopulation (NES for cMyc^+^lateCD23^int^ vs. cMyc^+^lateCD23^lo^ and cMyc^+^early vs. cMyc^+^lateCD23^int^; −1.17 and −2.37, respectively) (Fig. 2*D* and *SI Appendix*, Fig. S2*A*). These data indicated that the cMyc^+^ LZ B cell subpopulations emerge in the following order from the beginning of positive selection to DZ entry (cMyc^+^early → cMyc^+^lateCD23^int^ → cMyc^+^lateCD23^lo^), which in turn, indicates that the cMyc^+^early subpopulation has been more recently positively selected than the cMyc^+^late subpopulations.

To further test the hypothesis, we investigated if the size of each cMyc^+^ GC B cell subpopulation in silico would be commensurate to the in vivo results. To this end, we exploited an extended version of agent-based model for GC reactions (35) that includes features of multiple contacts between B and T cells to collect signals (36) (Fig. 2*E* and *SI Appendix*, Fig. S2 *B*–*E*). In this in silico model, the ratio of the percentage of recycling cells as DZ B cells (= centroblast [CB]) to the percentage of differentiating cells from the selected GC B cells as output cells was fixed to be 90%:10%. Under this setting, five different possible models (zero to four) for the functional interdependence of cMyc^+^ GC B cell subpopulations were tested. Temporal cellular dynamics of GC B cells, cMyc^+^ GC B cells, and cells in cMyc^+^ GC B cell subpopulations were obtained by in silico immunization (Fig. 2*E* and *SI Appendix*, Fig. S2 *B*–*E*), and the simulation results of each model reproduced the size of cMyc^+^ GC B cell subpopulations. Subsequently, quantitative model selection ranked the five models. The best-performing model (model 0) (Fig. 2*E*) was selected based on the lowest Akaike information criterion (AICc) value and residual sum of squares (RSS). Model 0 was considerably in accordance with the in vivo time-course results (i.e., cMyc^+^ LZ B cells progress from the cMyc^+^early subpopulation to the cMyc^+^late subpopulation that, in turn, becomes the cMyc^+^ DZ subpopulation). The in silico model also suggested that the cMyc^+^prePB subpopulation is derived from the cMyc^+^early subpopulation. Altogether, the data clearly indicated that cMyc^+^ GC B cell subpopulations represent those which emerge in a sequential order following the reception of cMyc-inducing signals.

### A Fraction of cMyc^+^ GC B Cells in Each Subpopulation Completes Mitosis in the LZ.

**Fig. 3.**
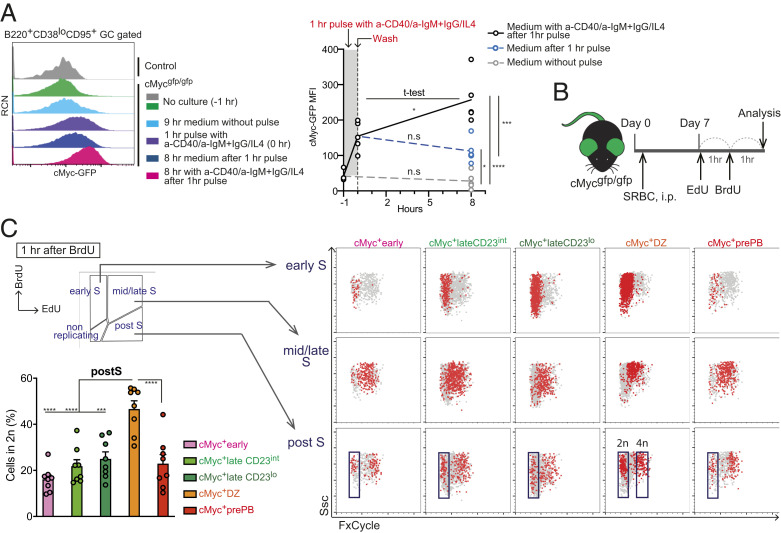
A fraction of cMyc^+^ GC B cells in each subpopulation completes mitosis in the LZ. (*A*) Representative flow cytometric histograms of cMyc-GFP vs. relative cell number (RCN; *Left*). cMyc-GFP MFI values of GC B cells in the indicated time points (*Right*). Each circle indicates one sample. (*B*) Experimental design for *C*. (*C*) Representative flow cytometric plots of a DNA dye, FxCycle vs. Ssc in the cMyc^+^ GC B cell subpopulations after 1-h BrdU incorporation. Diploid (2n) and tetraploid (4n) gates were determined by DNA contents that were visualized with FxCycle staining. Percentage of cells in the diploid gate of the post–S-gated cells is shown. This ratio indicates duration of S phase under this experimental setting. Splenic GC B cell response to SRBC on day 6 (*A*) or day 7 (*C*) in cMyc^gfp/gfp^ mice. Data are from a representative experiment containing five samples per condition of two experiments (*A*) or three experiments with eight mice (*C*). Error bars indicate SEM. Statistics were calculated with unpaired Student’s *t* test between samples taken at different time points (*A*) or with one-way ANOVA (*C*). n.s., not significant. *P < 0.05; ***P < 0.001; ****P < 0.0001.

Current knowledge does not provide information on whether and how long cMyc positivity induced by the engagement of BCR and CD40 is sustained in GC B cells. To mimic such positively selected B cells in vivo, we pulsed GC B cells for 1 h with anti-immunoglobulin M (IgM)/IgG F(ab’)_2_ (fragment antigen-binding) as a mimicry of BCR engagement, together with anti-CD40 antibody and IL-4 as a mimicry of TFH help, and investigated the level of cMyc in the cultured cells in the presence or absence of the stimulants for an additional 8 h. During the 8-h culture with the stimuli, cMyc-GFP mean fluorescence intensity (MFI) further increased compared with the MFI of the pulsed cells before the culture (Fig. 3*A*). Notably, in the absence of further stimuli, the cMyc-GFP MFI of the pulsed cells was maintained at a high level in these cells compared with the cells cultured in media for 9 h without any pulse (Fig. 3*A*). These data indicated that continuous stimulus is not required for the maintenance of cMyc expression in GC B cells for at least 8 h after cMyc induction. We concluded that cMyc^+^ LZ B cells can potentially complete at least one round of cell cycle in the LZ because the cell cycle of GC B cells takes 6 to 8 h (18, 37).

To assess the course of cell cycle progression within cMyc^+^ GC B cell subpopulations, we used the 5-ethynyl-2′-deoxyuridine (EdU)/5-bromo-2′-deoxyuridine (BrdU) dual-labeling technique. GC B cells were pulsed with EdU for 1 h and then labeled with BrdU for an additional 1 h, which enabled us to separate cells based on the cell cycle status (i.e., early synthesis [S] phase [EdU^neg^BrdU^+^], mid/late S phase [EdU^+^BrdU^+^], and post-S phase [EdU^+^BrdU^neg^]) (Fig. 3*B* and *SI Appendix*, Fig. S3*A*). When we closely analyzed the cell cycle status of each cMyc^+^ GC B cell subpopulation, we found varied dynamics between them: the percentage of cells in the nonreplicating gap 1 (G_1_) phase was greater in the cMyc^+^lateCD23^int^ subpopulation, whereas the percentage of cells in the post-S (gap 2 [G_2_]/mitosis [M]) was increased in the cMyc^+^early subpopulation (*SI Appendix*, Fig. S3*B*). On the other hand, the percentage of cells in the early S phase was increased within the cMyc^+^ DZ subpopulation (*SI Appendix*, Fig. S3*B*), suggesting that the speed of S-phase entry in this subpopulation was faster than cMyc^+^ LZ B cell subpopulations. A previous study has shown that the ratio of EdU^+^BrdU^+^/EdU^+^BrdU^neg^ × 1 h (interval time) can indicate duration of the S phase when the injection interval between EdU and BrdU is 1 h in dual-labeling experiments (38). Using this analysis, we found that the cMyc^+^prePB subpopulation exhibited the longest duration of the S phase, which was attributed to an increased percentage of cells in the mid-/late S phase (*SI Appendix*, Fig. S3 *B* and *C*). We also noted that the duration of the S phase for the cMyc^+^lateCD23^lo^ subpopulation was nearly twofold longer than those of the cMyc^+^early and cMyc^+^lateCD23^int^ subpopulations. However, the percentage of early S phase in the cMyc^+^lateCD23^lo^ subpopulation was higher than those in the cMyc^+^early and cMyc^+^lateCD23^int^ subpopulations (*SI Appendix*, Fig. S3 *B* and *C*). Considering the previous prediction that positively selected GC B cells return to the DZ before reaching the G_2_/M phase (35), this result may be partially attributed to the LZ to DZ transmigration of a fraction of cells within the cMyc^+^lateCD23^lo^ subpopulation during the S phase to become cMyc^+^ DZ cells. Consequently, the duration of the S phase for the cMyc^+^ DZ subpopulation determined in this way may be overestimated. These data were also supported by GSEA for cell cycle genes between cMyc^+^ LZ B cell subpopulations (*SI Appendix*, Fig. S3*D*).

Coupling a DNA-labeling dye to measure DNA contents with the EdU/BrdU dual-labeling technique allowed us to determine the cell cycle dynamics in detail. Analysis of EdU^+^BrdU^neg^ cells in the post-S phase (G_2_/M) of each cMyc^+^ GC B cell subpopulation revealed that all cMyc^+^ LZ B cell subpopulations contained post–S-phase cells and that a fraction of these cells had completed cytokinesis as indicated by the DNA content measurement (2n and 4n represented cells that were at G_1_ after cell division and were at G_2_/M, respectively) (Fig. 3*C*). This finding was further substantiated by the time-course examination of DNA contents of EdU^neg^BrdU^+^ cells (cells that had entered S phase) within each cMyc^+^ LZ B cell subpopulation. The analysis showed that DNA contents of EdU^neg^BrdU^+^ cells doubled up to the G_2_/M level (4n) from the early S phase (2n) (described as “S_1_” at 1 h after BrdU injection) 5 h after BrdU injection (i.e., within 4 h) (*SI Appendix*, Fig. S3 *E* and *F*). Histone H3 is specifically phosphorylated on Ser10 at prophase and is swiftly dephosphorylated by the end of mitosis. Therefore, phospho-histone H3 (pH3) at Ser10 is a specific marker for cells undergoing mitosis. In agreement with previous reports observing pH3^+^ mitotic GC B cells in the LZ (17, 19), we found pH3^+^GL7^+^ GC B cells in the CD35^hi^ LZ region (*SI Appendix*, Fig. S3*G*). Approximately 50% of pH3^+^ LZ B cells coexpressed cMyc at low levels (*SI Appendix*, Fig. S3*G*) in accordance with prior knowledge that levels of cMyc are drastically reduced during the brief period of mitosis (39). Notably, the percentage of cells that had completed cytokinesis (2n) of the post–S-phase gate had a nearly twofold increase in the cMyc^+^ DZ subpopulation compared with all the cMyc^+^ LZ B cell subpopulations (Fig. 3*C*). These data indicated that the G_2_/M phase progresses faster in the cMyc^+^ DZ subpopulation than the cMyc^+^ LZ B cell subpopulations and thus, that the tempo of cell cycle is likely to be different between the LZ and the DZ. Altogether, these data demonstrated that a fraction of cMyc^+^ LZ B cells completes cell cycle in each subpopulation in the LZ before migration to the DZ.

### Transit to a Subsequent cMyc^+^ LZ B Cell Subpopulation Can Be Accompanied by Cell Division.

**Fig. 4.**
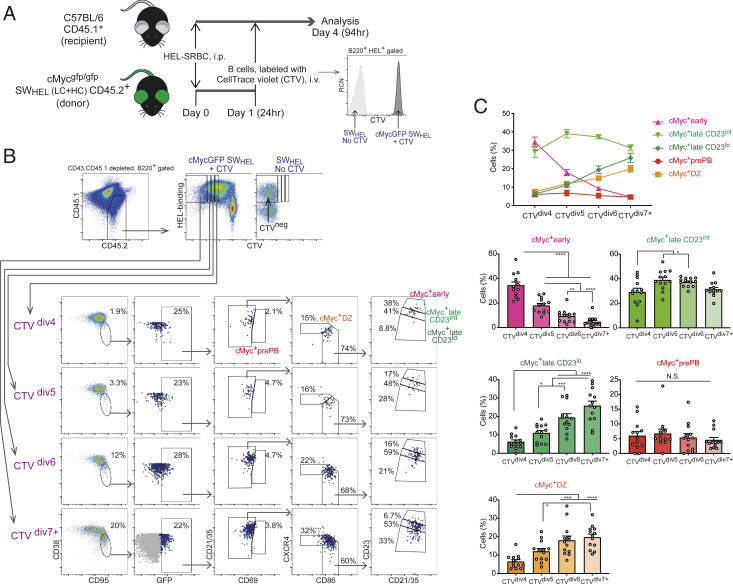
Transit to a subsequent cMyc^+^ LZ B cell subpopulation can be accompanied by cell division. (*A*) Experimental design for *B* and *C* with a representative flow cytometric plot of B cells used for adoptive transfer. Intraperitoneal (i.p.) and intravenous (i.v.) injections in mice. (*B*) Representative flow cytometric plots. SW_HEL_ cMyc^gfp/gfp^ donor-derived HEL-specific B220^+^ B cells were divided into four gates by cell division number based on dilution of CTV: CTV^div7+^, seven division and more; CTV^div6^, six division; CTV^div5^, five division; CTV^div4^, four division. cMyc-GFP^+^ GC B cells of each CTV gate were further resolved into the five subpopulations by the gating described in Fig. 1*D*. SW_HEL_ control mouse-derived GC B cells are shown in gray dots to identify the cMyc-GFP^neg^ population. (*C*) Percentages of cells of the five cMyc^+^ GC B cell subpopulations in each CTV gate. Pooled data from more than three experiments with 13 mice (*C*). Error bars indicate SEM. Statistics were calculated with one-way ANOVA. N.S., not significant. *P < 0.05; **P < 0.01; ***P < 0.001; ****P < 0.0001.

Given that a fraction of cMyc^+^ GC B cells in each LZ subpopulation completes mitosis, we aimed to examine whether the phenotypic change of cMyc^+^ LZ B cells is accompanied by cell division. For this purpose, B cells derived from Myc^gfp/gfp^ reporter mice crossed with SW_HEL_ mice that express the hen egg lysozyme (HEL)-specific immunoglobulin heavy chain knock-in and light chain transgene (HyHEL10 BCR) (40) were transferred into CD45.1^+^ congenic recipient mice. We studied the response of SW_HEL_ B cells to HEL after both donor and recipient mice were immunized with HEL-SRBC. One day after immunization, donor-derived CellTrace Violet (CTV)-labeled B cells were transferred into the immunized recipient mice (Fig. 4*A*). Seventy hours after the cell transfer, we analyzed the donor-derived HEL-specific B cells that had undergone cell division based on CTV dilution: CTV^div4^, CTV^div5^, CTV^div6^, and CTV^div7+^ (Fig. 4*B*). cMyc^+^ GC B cells located in these four CTV-based compartments were further resolved into the five cMyc^+^ GC B cell subpopulations (Fig. 4 *B* and *C*). Comparison of the percentage of each cMyc^+^ GC B cell subpopulation between the CTV compartments in sequence of division enabled us to calculate the proportion of cells transiting between subpopulation boundaries over time. The CTV^div4^ compartment consisted of a high proportion of cells in the cMyc^+^early subpopulation among total cMyc^+^ GC B cells; however, the percentage of cells in the subpopulation decreased nearly by half per round of cMyc^+^ B cell division: 34.5 ± 2.69% (CTV^div4^), 17.9 ± 1.71% (CTV^div5^), and 9.30 ± 1.45% (CTV^div6^). The percentage of cells comprising the cMyc^+^lateCD23^int^ subpopulation increased significantly between CTV^div4^ and CTV^div5^, and it decreased mildly after CTV^div5^: 29.2 ± 3.03% (CTV^div4^), 39.0 ± 2.26% (CTV^div5^), and 37.2 ± 0.994% (CTV^div6^). In contrast, the percentage of the cMyc+lateCD23^lo^ and cMyc^+^ DZ cells increased in every cell division: cMyc^+^lateCD23^lo^: 6.29 ± 1.02% (CTV^div4^), 11.0 ± 1.33% (CTV^div5^), and 19.4 ± 2.09% (CTV^div6^); cMyc^+^ DZ: 7.51 ± 1.33% (CTV^div4^), 11.9 ± 1.28% (CTV^div5^), and 14.8 ± 1.31% (CTV^div6^). Cells in the cMyc^+^prePB subpopulation maintained similar percentage throughout cell division (Fig. 4*C*). Collectively, these data suggested that cell division is correlated with the transit of a fraction of cells to a subsequent cMyc^+^ LZ B cell subpopulation.

### Permissive Selection Occurs at the Start of Positive Selection Followed by Affinity-Dependent Proliferation in the LZ.

**Fig. 5.**
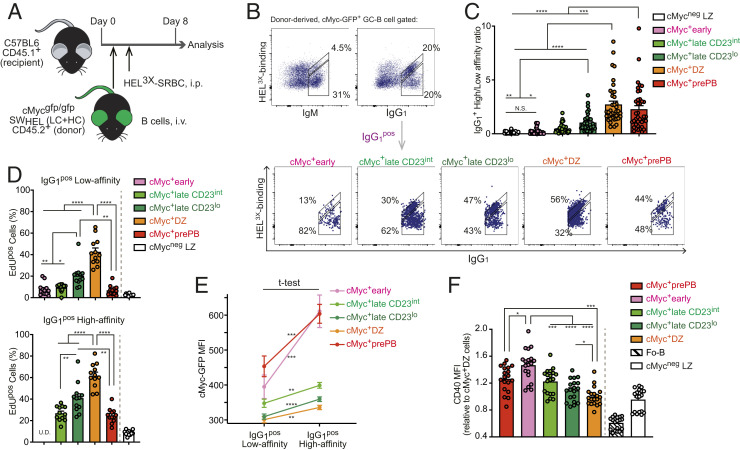
Permissive selection occurs at the start of positive selection, followed by affinity-dependent proliferation in the LZ. (*A*) Experimental design for *B*. (*B*) Representative flow cytometric plots. SW_HEL_ cMyc^gfp/gfp^ donor-derived cMyc^+^ GC B cells 8 d after HEL^3×^-SRBC immunization were divided into high-affinity (top diagonal gate) and low-affinity (bottom gate) gates based on binding ability of BCR (IgM or IgG_1_) to HEL^3×^ (*Upper*). Representative flow cytometric plots of HEL^3×^ binding vs. IgG_1_ expression in the cMyc^+^ GC B cell subpopulations (*Lower*). (*C*) IgG_1_^+^ high-/low-HEL^3×^ binding ratio in the cMyc^+^ GC B cell subpopulations. (*D*) Percentages of EdU^pos^ cells in the cMyc^+^ GC B cell subpopulations that were further divided based on the BCR affinity as shown in *B* 6 h after i.v. injection with EdU. Splenic GC B cell response to HEL^3×^-SRBC immunization on day 7. Due to the limitation of the number of high-affinity cells in the cMyc^+^early subpopulation, percentages of EdU^pos^ cells were unable to be determined (U.D.). (*E*) cMyc-GFP MFI values of the cMyc^+^ GC B cell subpopulations. SW_HEL_ cMyc^gfp/gfp^ donor-derived HEL-specific cells 8 d after HEL^3×^-SRBC immunization were divided into IgG_1_^pos^ high affinity and low affinity. Statistics calculated with unpaired Student’s *t* test between low- and high-affinity cells of each cMyc^+^ GC B cell subpopulation. (*F*) CD40 MFI values (relative to cMyc^+^ DZ values). Fo-B cells were defined as B220^+^CD38^hi^CD95^neg^CD23^hi^CD21/35^lo^ cells. Splenic GC B cell response to SRBC on day 7 in cMyc^gfp/gfp^ mice. Pooled data from more than three experiments with 38 mice (*C*), 12 mice (*D*), 18 mice (*E*), or 19 mice (*F*). Error bars indicate SEM. Statistics were calculated with one-way ANOVA. N.S., not significant. *P < 0.05; **P < 0.01; ***P < 0.001; ****P < 0.0001.

In the currently favored model, positive selection occurs in an affinity-dependent manner in the LZ (7, 8). To investigate if BCR affinity differs between cMyc^+^ GC B cell subpopulations, we endeavored to measure it. B cells derived from Myc^gfp/gfp^ SW_HEL_ mice were transferred into CD45.1^+^ congenic recipient mice, followed by immunization with an HEL mutant protein, HEL^3×^ conjugated to SRBC (Fig. 5*A*). HyHEL10 BCR binds to HEL with high affinity (2 × 10^10^ M^−1^), whereas it binds to HEL^3×^ with an affinity 13,000-fold lower (41), which allows us to assess affinity maturation in mice after HEL^3×^-SRBC immunization. As previously shown, donor cells hardly respond to SRBC (42). Eight days after immunization, high-affinity SW_HEL_ B cell clones bound to HEL^3×^ more intensely (top diagonal gate), whereas low-affinity clones bound to it less intensely (bottom gate) (Fig. 5*B*). Flow cytometric analysis showed that high-affinity clones were almost exclusively enriched within class-switched IgG_1_^+^ cells but not in IgM^+^ cells (Fig. 5*B*). Thereafter, we restricted the analysis to IgG_1_^+^ cells, which also ensures that IgM^+^ naïve B cells were excluded from the analysis. We looked at the ability of cMyc^+^ GC B cell subpopulations to bind HEL^3×^ and found that the cMyc^+^early subpopulation predominantly contained lower-affinity clones, whereas the cMyc^+^prePB subpopulation comprised higher-affinity cells (Fig. 5*C*). To ensure that the cMyc^+^early subpopulation did not contain a significant fraction of recent GC entrants, we sorted this subpopulation derived from cMyc^gfp/gfp^ reporter mice 10 d after immunization with NP-CGG by flow cytometry and investigated the number of mutations in the NP-binding IgG_1_ VH186.2 gene sequence. Only 8.6% of the total sequenced IgG_1_^+^ clones in the cMyc^+^early subpopulation were detected as unmutated (162 clones sequenced; results from pooled three independent sorting; unmutated clone number/total sequenced: sort#1, 6/45; sort#2, 3/56; sort#3, 5/61). This indicated that the cMyc^+^early subpopulation is mainly derived from GC residents that have undergone somatic hypermutation rather than from cells that have recently entered the GC, in keeping with the previous observation that only B cell clones with a competitive advantage in affinity can join preexisting GCs (43). The ratio of IgG_1_^+^ high/low HEL^3×^ binding cells was increased by approximately fourfold when the values were compared between the cMyc^+^early and the cMyc^+^lateCD23^lo^ subpopulations (Fig. 5*C*). Although it is generally assumed that only high-affinity clones are actively positively selected from the beginning of the process, our data revealed that positive selection starts permissively to retain lower-affinity cells. Cells in cMyc^+^ GC B cell subpopulations were not apoptotic despite the fact that most of the cells in the cMyc^+^early and cMyc^+^lateCD23^int^ subpopulations carried low-affinity BCR (Fig. 5*C* and *SI Appendix*, Fig. S4*A*). In agreement with previous reports showing that high-affinity GC B cells proliferate more than low-affinity ones (12, 25), a greater percentage of high-affinity cells incorporated EdU than low-affinity ones within each cMyc^+^ GC B cell subpopulation (Fig. 5*D*). This could not be determined for the cMyc^+^early subpopulation due to limitation in high-affinity cell numbers at the experimental time point (Fig. 5*D*). As a result of preferential proliferation of higher-affinity clones, BCR affinity was enhanced in the LZ, while cMyc^+^ LZ B cells were phenotypically changing to cMyc^+^ DZ B cells (i.e., from cMyc^+^lateCD23^int^ to cMyc^+^lateCD23^lo^ and from cMyc^+^lateCD23^lo^ to cMyc^+^ DZ) (Fig. 5 *B* and *C*). Because the relationship between the cMyc^+^prePB and the rest of the cMyc^+^ LZ B cell subpopulations was not well defined (described below), we excluded the cMyc^+^prePB subpopulation from the model. In summary, these data suggest a revised model for the positive selection process: GCs are permissive to selecting lower-affinity cells at the start of positive selection, followed by affinity-dependent proliferation in the LZ. Clonal diversity is ensured by protecting lower-affinity cells from undergoing apoptosis, while the affinity of future DZ entrants is increased in the LZ before DZ entry.

### Levels of cMyc Expression Reinforce the Potential for cMyc^+^ GC B Cell Subpopulations to Emerge Sequentially.

A previous report has demonstrated that the level of cMyc protein is highest in B cells that have just received cMyc-inducing signals (44). Hence, the level of cMyc expression is a good indicator for how long the cells have received cMyc-inducing signals. We found that the cMyc-GFP MFI was highest in the cMyc^+^early subpopulation and decreased in the following order: cMyc^+^early, cMyc^+^lateCD23^int^, cMyc^+^lateCD23^lo^, and cMyc^+^ DZ subpopulations for both low- and high-affinity cells (*SI Appendix*, Fig. S4*B*). This result reinforced our hypothesis that the cMyc^+^early subpopulation emerges earliest, and the cMyc^+^lateCD23^int^ and cMyc^+^lateCD23^lo^ subpopulations follow it in this order. Previous work showed that the level of cMyc protein in GC B cells is also dependent on the amount of T cell help (45), which in turn, is dependent on BCR affinity. As expected, high-affinity cells expressed more cMyc protein than low-affinity cells when the comparison was made within the same cMyc^+^ GC B cell subpopulation (Fig. 5*E*). Despite that, the low-affinity cells of the cMyc^+^early subpopulation exhibited a similar level of cMyc-GFP expression compared with the high-affinity cells of the cMyc^+^lateCD23^int^ subpopulation and even higher than that of the high-affinity cells in the cMyc^+^lateCD23^lo^ and cMyc^+^ DZ subpopulations (Fig. 5*E*). A similar observation was made in mice immunized with SRBC, in which the cMyc^+^early subpopulation exhibited higher cMyc-GFP MFI compared with cMyc^+^late and cMyc^+^ DZ subpopulations regardless of its larger proportion of low-affinity clones (*SI Appendix*, Fig. S4*C*). In order to understand how cells in the cMyc^+^early subpopulation can be positively selected despite the enrichment of low-affinity clones, we investigated the expression of CD40 that is a key molecule for cMyc induction (46). In cMyc^+^ GC B cell subpopulations derived from cMyc^gfp/gfp^ mice on day 7 of the response to SRBC, cells in the cMyc^+^early subpopulation expressed higher surface levels of CD40 than cMyc^neg^ LZ B cells and any other cMyc^+^ GC B cell subpopulation (Fig. 5*F* and *SI Appendix*, Fig. S4*D*). These data suggested that CD40 is up-regulated in low-affinity LZ B cells before and/or during receiving signals from TFH to allow them to receive increased T cell help and pass a threshold (i.e., cMyc induction) for positive selection.

Cells in the cMyc^+^prePB subpopulation expressed cMyc as high as cells in the cMyc^+^early subpopulation (Fig. 5*E* and *SI Appendix*, Fig. S4 *B* and *C*), and also, the cMyc^+^prePB subpopulation was more closely related to the cMyc^+^early subpopulation than any of the other cMyc^+^ LZ B cell subpopulations according to the 3D PCA plots (Fig. 2*C*). These data together with in silico models (Fig. 2*E* and *SI Appendix*, Fig. S2 *B*–*E*) suggested the possibility that the cMyc^+^prePB subpopulation may be selectively derived from high-affinity cells within the cMyc^+^early subpopulation. However, cells in the cMyc^+^prePB subpopulation were comparatively more enriched in the cMyc^+^AP4^+^ LZ positively selected GC B cell gene signature than the cMyc^+^early subpopulation (Fig. 2*D*). Thus, we currently cannot exclude the possibility that cMyc^+^prePB subpopulation may be generated independently of the cMyc^+^early subpopulation.

Altogether, these results strongly reinforced our hypothesis that cMyc^+^ GC B cell subpopulations represent those which emerge in a sequential order following cMyc-inducing signals. The process of positive selection starts in the cMyc^+^early subpopulation, which is predominantly composed of low-affinity clones and proceeds through preferential proliferation of high-affinity clones in the subsequent cMyc^+^late subpopulations to outcompete the low-affinity ones in the LZ.

### Specific cMyc^+^ GC B Cell Subpopulations Contain MBC and PB/PC Precursors.

**Fig. 6.**
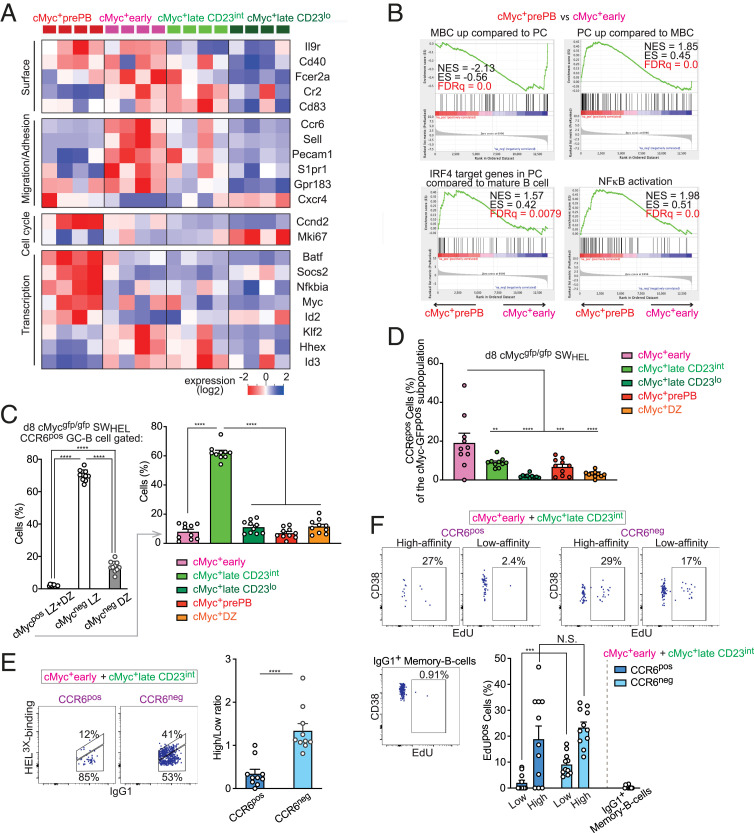
Specific cMyc^+^ GC B cell subpopulations contain MBC and PB/PC precursors. (*A*) Heat map for selected genes known to be associated with PB/PC or MBC differentiation. Only genes that have passed the threshold of *P* ≤ 0.04 by multigroup comparison are shown. (*B*) GSEA of differential gene expression in the cMyc^+^prePB subpopulation vs. the cMyc^+^early subpopulation for gene sets: “up-regulated in MBC compared with PC,” “up-regulated in PC compared with MBC,” “IRF4 target in PC compared with mature B cell,” and “NFκB activation.” Nominal enrichment score (NES), enrichment score (ES) and false discovery rate (FDR) q values. (*C*) Percentages of cells within the indicated populations after gating on CCR6^pos^ GC B cells as shown in *SI Appendix*, Fig. S5*C* (*Left*). Percentage of cells within the cMyc^+^ GC B cell subpopulations after gating on CCR6^pos^ cMyc^+^ GC B cells (*Right*). (*D*) Percentage of CCR6^pos^ cells in the cMyc^+^ GC B cell subpopulations after gating as shown in *SI Appendix*, Fig. S5*C*. (*E*) Representative flow cytometric plots of HEL^3×^ binding vs. IgG_1_ expression in CCR6^pos^ and CCR6^neg^ cells of the cMyc^+^early and cMyc^+^lateCD23^int^ combined populations (*Left*). IgG_1_^pos^ high-/low-HEL^3×^ binding ratio in the indicated cell populations as gated in *Left* (*Right*). (*F*) Representative flow cytometric plots of CD38 vs. EdU incorporation in CCR6^pos^ and CCR6^neg^ cells of the cMyc^+^early and cMyc^+^lateCD23^int^ combined populations that were further divided based on the BCR affinity as shown in *E* and IgG_1_^pos^ MBCs 6 h after intravenous injection with EdU. Percentages of EdU^pos^ cells in the indicated cell populations (*Lower Right*). SW_HEL_ cMyc^gfp/gfp^ donor B cells on day 8 (*C*–*E*) or day 7 (*F*) after HEL^3×^-SRBC immunization. Pooled data from two experiments with 10 mice (*C*–*E*) or 11 mice (*F*). Error bars indicate SEM. Statistics were calculated with one-way ANOVA (*C* and *D*) or unpaired Student’s *t* test (*E* and *F*). N.S., not significant. **P < 0.01; ***P < 0.001; ****P < 0.0001.

We next examined transcriptomic profiles to investigate whether cMyc^+^ LZ B cell subpopulations are associated with B cell differentiation. First, within the DEGs identified by multigroup comparison (*P* ≤ 0.04) between the four cMyc^+^ LZ B cell subpopulations from bulk RNAseq data, we analyzed those genes that are known to be associated with PB/PC or MBC differentiation (23, 47–49). Cells in the cMyc^+^prePB subpopulation had increased expression of PC-associated genes, including *Batf*, *Socs2*, *Nfkbia*, *Myc*, *Id2*, *Cd40*, and *Ccnd2*, but reduced expression of *Id3*, *Sell*, *Cr2*, *Cd83*, and *Ccr6*, which is in agreement with the low expression of these genes in PCs (23, 47, 49) (Fig. 6*A*). The increased expression of *Nfkbia* in the cMyc^+^prePB subpopulation was an indication of enhanced NF-κB signaling, an essential pathway for the differentiation of PB/PC from GC B cells (28). Then, we performed GSEA on the cMyc^+^prePB subpopulation, which displayed significant enrichment of a PC gene signature and the expression of IRF4 target genes in PCs (Fig. 6*B*). These results suggested that the cMyc^+^prePB subpopulation contains PB precursors. Although the gene expression of *Prdm1* or *Irf4* was not significantly up-regulated in this subpopulation, we found that cMyc^+^prePB subpopulation had an increased percentage of Blimp1-GFP^+^ cells (50) and relatively higher levels of IRF4 compared with the other cMyc^+^ GC B cell subpopulations (*SI Appendix*, Fig. S5 *A* and *B*). Surprisingly, genes associated with MBCs such as *Il9r*, *Ccr6*, *Sell*, *Pecam1*, *S1pr1*, *Gpr183*, *Klf2*, and *Hhex* (47, 48, 51) were up-regulated in the cMyc^+^early subpopulation (Fig. 6*A*), which was accompanied by a significant enrichment of an MBC gene signature in this subpopulation (Fig. 6*B*).

MBC precursors are reported to be quiescent and express lower-affinity BCR (24–26), whereas cMyc^+^ GC B cells have been known to be proliferative and carry higher-affinity BCR (4, 5). Hence, it has been counterintuitive to link MBC precursors to positively selected cMyc^+^ GC B cells. However, the analysis of the bulk RNAseq data showed a significant enrichment of genes associated with MBCs in the cMyc^+^early subpopulation. We thereby investigated whether MBC precursors defined by CCR6 expression (25) were present within cMyc^+^ GC B cell subpopulations. To this end, we adoptively transferred B cells from SW_HEL_ Myc^gfp/gfp^ mice and first examined if donor-derived CCR6^pos^ GC B cells expressed cMyc 8 d after HEL^3x^-SRBC immunization. We found CCR6^pos^ cells within cMyc^+^ LZ B cells (2.0 ± 0.17% of total CCR6^pos^ GC B cells) (Fig. 6*C*) despite the enrichment of CCR6^pos^ GC B cells within cMyc^neg^ LZ B cells (Fig. 6*C* and *SI Appendix*, Fig. S5*C*). We next examined which cMyc^+^ GC B cell subpopulation represented CCR6^pos^ GC B cells and found that the cMyc^+^lateCD23^int^ subpopulation comprised over 60 of the cMyc^+^ CCR6^pos^ GC B cells (62 ± 1.6%) (Fig. 6*C*). In contrast, the cMyc^+^ DZ subpopulation was not enriched with CCR6^pos^ GC B cells (Fig. 6*C* and *SI Appendix*, Fig. S5*C*). These results are concordant with the previous observation that CCR6^pos^ GC B cells are predominantly located in the LZ (25). When the proportion of CCR6^pos^ B cells within each cMyc^+^ GC B cell subpopulation was determined, the cMyc^+^early subpopulation contained the highest percentage of CCR6^pos^ GC B cells (19 ± 4.3% in SW_HEL_Myc^gfp/gfp^ mice and 18 ± 2.6% in Myc^gfp/gfp^ mice) (Fig. 6*D* and *SI Appendix*, Fig. S5*D*, respectively). Hence, cMyc^+^CCR6^pos^ MBC precursors were present, and these cells were mostly contained in the cMyc^+^early and cMyc^+^lateCD23^int^ subpopulations. Accordingly, we performed further analysis of MBC precursors using these combined subpopulations. The cMyc^+^CCR6^pos^ MBC precursors were predominantly composed of low-affinity clones (Fig. 6*E*) in agreement with the previous finding that MBCs are mostly derived from low-affinity cells (24, 25). Indeed, higher-affinity clones became dominant in GCs over time (*SI Appendix*, Fig. S5*E*), and the percentage of MBC precursors found within cMyc^+^ GC B cell subpopulations 15 d after HEL^3x^-SRBC immunization was highly reduced (*SI Appendix*, Fig. S5*F*) compared with those after 8 d after immunization (Fig. 6*D*). This finding is consistent with the knowledge that MBCs mostly arise at earlier stages of the GC reaction (52).

Next, we investigated if the cMyc^+^CCR6^pos^ MBC precursors were less proliferative as previously reported for MBC precursors (25, 26). To examine the proliferation status of CCR6^neg^ GC B cells and CCR6^pos^ GC B cells within the low- and high-affinity cMyc^+^early and cMyc^+^lateCD23^int^ combined subpopulations, we performed EdU incorporation assays. Within the cMyc^+^ GC B cell combined subpopulations, a significantly lower percentage of low-affinity cMyc^+^CCR6^pos^ MBC precursors incorporated EdU compared with low-affinity cMyc^+^CCR6^neg^ GC B cells (Fig. 6*F*). These data demonstrated that low-affinity cMyc^+^CCR6^pos^ MBC precursors are less proliferative than the rest of the cMyc^+^ GC B cells (Figs. 5*D* and 6*F*). In summary, we identified CCR6^pos^ MBC precursors within cMyc^+^ GC B cells. This identification provides a biological rationale for permissive positive selection, by which antigen-specific MBCs bearing low-affinity BCR can be efficiently produced.

## Discussion

This study redefines GC B cell–positive selection as a dynamic process that ensures maintenance of a broad range of affinities in GCs. We found that affinity-dependent cell division occurred in the LZ and therefore, that such process is not restricted to the DZ. Newly positively selected LZ B cells form the cMyc^+^early subpopulation, which consists predominantly of low-affinity cells that are protected from apoptosis. These cMyc^+^ LZ B cells give rise to subsequent cMyc^+^late subpopulations in which high-affinity cells divide more than low-affinity cells. Such an affinity-based proliferation in the LZ allowed the enrichment of high-affinity clones before DZ migration. This mode of positive selection can be advantageous for retaining varied affinity clones in GCs, which ultimately may contribute to the production of GC-derived MBCs (*SI Appendix*, Fig. S6).

Cells in each cMyc^+^ LZ B cell subpopulation are proliferative, although their cell cycle dynamics varied in each subpopulation. This proliferative status of cMyc^+^ LZ B cell subpopulations is consistent with previous observations of a fraction of dividing LZ B cells made by histology, two-photon microscopy, and flow cytometry (7, 16–19) and also with dividing LZ B cells (S/G_2_/M) being located at the border of LZ/DZ (53). Although these results may also reflect the arrival of dividing DZ B cells to the LZ, our flowcytometric data demonstrated that a fraction of positively selected LZ B cells initiated and completed cell division locally. Immunofluorescence results showed that mitotic pH3^+^cMyc^+^ GC B cells exist in the LZ (*SI Appendix*, Fig. S3*G*), and accordingly, a fraction of cMyc^+^ GC B cells divides in the LZ. However, we are currently unable to identify spatial distribution of cells in each cMyc^+^ subpopulation due to technical limitations. Recently, a proliferative CXCR4^+^CD83^+^ GC B cell subset, “grey zone (GZ) B cells,” has been discovered with a new gating strategy, and this subset is transcriptionally distinct from the rest of GC B cells, namely CXCR4^neg^CD83^+^ LZ B cells and CXCR4^+^CD83^neg^ DZ B cells (54). Approximately one-third and two-thirds of GZ B cell subset are LZ B cells (CXCR4^neg/+^CD83^+^) and DZ B cells (CXCR4^+^CD83^neg/+^), respectively, when cells are delineated using the conventional gating strategy (54). A large fraction of cells in the cMyc^+^ subpopulations were shown to be located in the CXCR4^+^CD86^+^ region (Fig. 2*A*). Because it is most likely that this region is equivalent to the CXCR4^+^CD83^+^ GZ region, the GZ B cell subset perhaps includes most cells from the cMyc^+^ subpopulations. Approximately 3 to 7% of GC B cells are cMyc^+^ LZ B cells, which is proportionally much smaller than constantly dividing DZ B cells. Therefore, cell division of a small number of cMyc^+^ LZ B cells may be underestimated particularly when GC B cells are treated with potent exogenous cell cycle inducers (45).

Considering the cell number and transition of high-/low-affinity ratio between the three cMyc^+^ LZ B cell subpopulations (cMyc^+^early, cMyc^+^lateCD23^int^, and cMyc^+^lateCD23^lo^), some clones bearing high-affinity BCR perhaps divide more than once in the LZ. This is consistent with our finding showing that a fraction of cells completes cell division within a specific cMyc^+^ LZ B cell subpopulation. We revealed that GC B cells can retain cMyc expression in the absence of continued stimulation for at least 8 h after reception of cMyc-inducing signals (Fig. 3*A*). Given that GC B cells divide every 6 to 8 h (18, 37), multiple rounds of division of LZ B cells are theoretically possible. Our data suggested that the phenotypic change of cMyc^+^ LZ B cells can be accompanied by cell division. However, given that the number of cell division of cMyc^+^ LZ B cells with low-affinity BCR is fewer than those with high-affinity BCR, we assume that the phenotype associated with the cMyc^+^ LZ B cell subpopulations is perhaps altered in the absence of cell division as well. Due to limitations of the current technology, we are so far unable to determine whether a cell divides in every cMyc^+^ LZ B cell subpopulation and accordingly, how many times cMyc^+^ LZ B cells divide before migrating to the DZ. Further investigation is required for the elucidation of these points.

Abrogation of FoxO1 and CXCR4, key molecules to maintain the DZ program and to define DZ positioning of GC B cells, results in generating DZ-null GCs according to surface marker profile (19–21, 55). Notably, the effect caused by the loss of either molecule on cell cycle status is minimal, indicating that neither DZ access nor the FoxO1-governing DZ program are required for cell proliferation (19, 20, 55). These data suggest that the DZ is not the only site for cell proliferation in agreement with our data and with earlier observations of proliferative cells in the LZ (7, 16–18, 37). Since *Foxo1*-null LZ-like B cells are defective in antigen presentation due to a significantly lowered expression of BCR compared with control LZ B cells (21), positive selection in these mice is impaired. As a result, a reduced percentage of high-affinity clones or antigen-specific cells is observed in *Foxo1*-deficient mice compared with control mice (20, 55). Nonetheless, Sander et al. (20) found a similar percentage of high-affinity clones between IgM^+^ NP-specific *Foxo1*-null GC B cells and control IgM^+^ NP-specific GC B cells. It is thus tempting to speculate that this finding may be attributable to proliferation of positively selected LZ B cells independently of the DZ proliferation program.

It remains unclear how the cMyc^+^prePB subpopulation is generated. The cMyc^+^prePB subpopulation expressed lower levels of CD40 than the cMyc^+^early subpopulation (Fig. 5*F* and *SI Appendix*, Fig. S4*D*). This result can be interpreted as that CD40 internalization had started more recently in the cMyc^+^early subpopulation than the cMyc^+^prePB subpopulation (i.e., the former subpopulation is an ascendant subpopulation of the latter). GSEA results supported this interpretation because the signature of genes up-regulated by CD40 signaling was found to be enriched in the cMyc^+^prePB subpopulation compared with the cMyc^+^early subpopulation. Moreover, the in silico model also indicated that the cMyc^+^prePB subpopulation is derived from the cMyc^+^early subpopulation (Fig. 2*E*). In such a case, cells in the cMyc^+^prePB subpopulation are likely to be derived from high-affinity cells within the cMyc^+^early subpopulation.

MBCs are enriched with low-affinity clones and appear to require only minimal levels of signals derived from TFH (24, 25). Therefore, it is broadly assumed that MBCs arise from nonpositively selected LZ B cells as the TFH help provided is below a threshold for positive selection (24). To understand the origin of MBCs, intensive analysis using scRNAseq identified a pre-MBC subset in virus-infected mouse spleens (51) and human tonsils (56). The pre-MBC subsets are characterized by the expression of CCR6 in the both cases, and the mouse pre-MBC subset has relatively higher *Mki67* expression than MBC subsets (51). This finding suggests that some CCR6^+^ pre-MBCs are able to divide before differentiation into MBCs. Here, our data indicate that MBCs can emerge from CCR6^+^cMyc^+^ GC B cells that are still dividing. Our flow cytometric analysis and GSEA results revealed that cells within the combined subpopulations of cMyc^+^early and cMyc^+^lateCD23^int^, which are mostly composed of low-affinity clones, are a source of CCR6^+^ MBC precursors (Fig. 6 *A*–*D* and *SI Appendix*, Fig. S5 *C* and *D*). Low-affinity CCR6^+^cMyc^+^ MBC precursors were less proliferative compared with low-affinity CCR6^neg^cMyc^+^ LZ B cells within the combined subpopulations (Fig. 6*F*). Proliferation of high-affinity CCR6^+^cMyc^+^ MBC precursors was comparable with that of high-affinity CCR6^neg^cMyc^+^ LZ B cells within the same combined subpopulations (Fig. 6*F*); however, this does not exclude a possibility that a small fraction of high-affinity MBCs can emerge from CCR6^+^cMyc^+^ LZ B cells carrying high-affinity BCR. For the MBC fate decision in cMyc^+^ GC B cells, coexpression of cMyc-interacting proteins perhaps plays a key role. We recently discovered that cMyc binds to the transcription factor MIZ1 to restrict positively selected GC B cells from forming MBCs and favor expansion of GC B cells (57). Hhex, an important transcription factor for MBC differentiation (51), can interact with cMyc to decrease its activity, including cell proliferation and metabolism in tumors (58). Further investigation on interplays between transcription factors is required to understand underlying mechanisms of MBC differentiation from cMyc^+^ GC B cells.

Although a proportion of low-affinity cells has been found in GCs (14, 15), it was unknown how those cells are maintained. We showed that low-affinity cells are selected at first, and thus, those cells escape apoptosis in agreement with cMyc^+^ GC B cells being protected from cell death (6). Interestingly, the earliest positively selected subpopulation (cMyc^+^early) up-regulated CD40 on the surface compared with cMyc^neg^ LZ B cells, presumably to compensate insufficiency of T cell help due to their low-affinity BCR. Elevated levels of CD40 expression perhaps enable future cMyc^+^early cells to pass the positive selection threshold (i.e., cMyc induction) because acquisition of T cell help is required for optimal cMyc expression (46). In vitro analysis of CD8^+^ T cells revealed that the levels of cMyc protein within the cMyc^+^ compartment are identical regardless of the ligand affinities for the TCR if the ligand is abundantly available, suggesting that TCR signaling functions mostly as an on/off switch. In contrast, the strength of cytokine signaling is essential for maintaining and fine-tuning the levels of cMyc protein in CD8^+^ T cells (59). GC B cells may also require an extra regulatory mechanism for cMyc expression during positive selection; thus, cosignaling molecules and/or cytokines may potentiate CD40 expression regardless of BCR affinity to allow cells to receive sufficient amount of T cell help. This may occur through signals derived from FDCs and/or various TFHs that can differentially regulate GC B cells (60). Experiments using mouse and human B cells suggest that IL-4 and B cell activating factor (BAFF) (61, 62) can play important roles in the up-regulation of CD40 on B cells; however, further experimental insight is required.

In summary, we have identified and characterized temporally and functionally distinct cMyc^+^ GC B cell subpopulations. The analysis elucidated that positive selection is a dynamic process associated with GC B cell differentiation from the cMyc^+^ LZ B cell compartment. Such a dynamic positive selection process may provide optimized differentiation for MBCs that are required for broad protection from infectious agents by guaranteeing the survival and maintenance of lower-affinity cells in the GC reaction. Moreover, the subdivision of the cMyc^+^ compartment provides an invaluable foundation for the study of the molecular mechanisms of selection and differentiation of cMyc^+^ GC B cells, which may aid to design vaccines. In-depth knowledge of cMyc^+^ GC B cells can be also useful in investigating roles of molecules that cooperatively function with cMyc as this protein is a well-known protooncogene. In the long term, this direction of study can lead to better understanding of cMyc-induced lymphomagenesis.

## Materials and Methods

### Mice.

SW_HEL_ mice (40), cMyc^gfp/gfp^ mice (27), prdm1^gfp/+^ (50), and B6SJL CD45.1 mice were maintained on C57BL/6J background. Mice were bred and kept under specific pathogen-free conditions at the Francis Crick Institute Biological Research Facility in accordance with guidelines set by UK Home Office and the Francis Crick Institute Ethical Review Panel.

### Flow Cytometry and Cell Sorting.

Multicolor flow cytometry for analyses or for cell sorting was performed on LSR Fortessa or FACSAria (BD Biosciences), and data were analyzed using FlowJo v.10.5.3 software.

### scRNAseq and RNAseq.

The samples were sequenced using the HiSeq4000 system (Illumina). Additional information can be found in *SI Appendix*, *SI Materials and Methods*.

## Supplementary Material

Supplementary File

## Data Availability

RNAseq data have been deposited in the National Center for Biotechnology Information Gene Expression Omnibus (accession nos. GSE129857, GSE147098, and GSE146987). All study data are included in the article and supporting information.
