# Supplementary material for: Permissive selection followed by affinity-based proliferation of GC light zone B cells dictates cell fate and ensures clonal breadth
Source: Proc Natl Acad Sci U S A. 2021 Jan 8;118(2):e2016425118. doi: 10.1073/pnas.2016425118 (PMC7812803; doi:10.1073/pnas.2016425118)
Supplement: Supplementary File [file pnas.2016425118.sapp.pdf]

PNAS
www.pnas.org

Supplementary Information for

Permissive selection followed by affinity-based proliferation of GC light zone B cells dictates cell fate and ensures clonal breadth

Rinako Nakagawa[a,1], Amparo Toboso-Navasa[a,2], Marta Schips[b,2], George Young[c], Leena Bhaw-Rosun[d], Miriam Llorian-Sopena[e], Probir Chakravarty[e], Abdul Karim Sesay[d], George Kassiotis[c], Michael Meyer-Hermann[b,f] and Dinis Pedro Calado[a,g,1]

[a]Immunity and Cancer Laboratory, Francis Crick Institute, London NW1 1AT, United Kingdom

[b]Department of Systems Immunology and Braunschweig Integrated Centre for Systems Biology, Helmholtz Centre for Infection Research, 38106 Braunschweig, Germany

[c]Retroviral Immunology Laboratory, Francis Crick Institute, London NW1 1AT, United Kingdom

[d]Advanced Sequencing Laboratory, Francis Crick Institute, London NW1 1AT, United Kingdom

[e]Bioinformatics and Biostatistics Laboratory, Francis Crick Institute, London NW1 1AT, United Kingdom

[f]Institute for Biochemistry, Biotechnology and Bioinformatics, Technische Universität Braunschweig, 38106 Braunschweig, Germany

[g]Peter Gorer Department of Immunobiology, School of Immunology & Microbial Sciences, King's College London, London WC2R 2LS, United Kingdom

[1]To whom correspondence may be addressed: rinako.nakagawa@crick.ac.uk or dinis.calado@crick.ac.uk.

[2]A.T.-N. and M.S. contributed equally to this work.

This PDF file includes:

- SI Materials and Methods
- Figures S1 to S6
- SI References

Supplementary Information Text

SI Materials and Methods

Immunization and adoptive transfer. Mice were immunized intraperitoneally with 100 µg of NP-CGG in Imject Alum Adjuvant (Thermo Fisher Scientific) or 2×10^8 SRBCs. For HEL-specific GC responses, splenic B-cells from donor mice were enriched by CD43 depletion using MACS LS Columns (Miltenyi Biotec) and 5×10^4 to 1×10^5 HEL-specific B-cells were transferred into B6SJLCD45.1$^+$ hosts intravenously. Mice were immunized intraperitoneally on the same day of cell transfer or one day later with 2×10^8 SRBCs conjugated with HEL$^{3\times}$ (1) (a gift from R.Brink, Garvan Institute of Medical Research/University of New South Wales). For CTV stained cell transfer, splenic B-cells from donor mice were enriched by CD43 depletion using MACS LS Columns, followed by staining with 5µM CellTrace Violet (Thermo Fisher Scientific) in PBS for 15 min at RT before cell transfer. The cells were washed as described by the manufacturer.

Antibodies, flow cytometry and cell sorting. PBS + 2% FBS was used as flow cytometry buffer. Harvested splenic B-cells were enriched by CD43/IgD/CD4 depletion using MACS LS Columns to remove CD43$^+$ PBs (2), non-B-cells and follicular-B cells. For HEL-specific GC responses, splenic B-cells were enriched by CD43 depletion, followed by CD45.1 depletion using MACS LS Columns to remove recipient-derived cells. Single-cell suspensions were prepared and stained for the antibodies specific for B220 (RA3-6B2; BioLegend/BD Biosciences), CD19 (1D3; BD Biosciences), CD38 (90; BD Biosciences/BioLegend/Thermo Fisher Scientific), CD95 (Jo2; BD Biosciences), CD86 (GL1; BioLegend), CXCR4 (2B11; Thermo Fisher Scientific), CD69 (H1.2F3; BioLegend/BD Biosciences), CD21/35 (7E9 or 7G6; BioLegend/BD Biosciences), CD23 (B3B4; BioLegend), GL7 (GL7; Thermo Fisher Scientific), IgG1 (A85-1; BD Biosciences), IgM (II/41; BD Biosciences), IgD (11-26c.2a; BioLegend), CD45.2 (104; BD Biosciences), CD4 (GK1.5; BioLegend), CD40 (3/23; BD Biosciences), CD196 (140706; BD Biosciences), CD138 (281-2; BD BioLegend/Biosciences) and CD44 (IM7; BioLegend). HEL-binding cells were stained with HEL (50 ng/ml), followed by HyHEL9 Alexa Fluor 647. For intracellular staining, cells were fixed and permeabilized using a Fixation/Permeabilization Solution Kit (BD Biosciences) and stained with anti-cMyc antibody (Y69; Abcam) or isotype control (EPR25A; Abacm), or anti-active caspase 3 antibody (C92-605; BD Biosciences). For Irf4 staining, cells were further permeabilized with 0.2% Triton-X 100 and followed by staining with

anti-Irf4 antibody (3E4; Thermo Fisher Scientific) or isotype control (eBRG1; Thermo Fisher Scientific). To achieve consistent flow cytometric gates for the delineation of $cMyc^{+}$ GC-B-cell subpopulations as described in Fig. 1D, splenic GC-B-cells from $cMyc^{gfp/gfp}$ mice 7 days after SRBC immunization were surface stained, if necessarily fixed and permeabilized to match with an experiment, and used to set the gates in every experiment.

***Ex vivo* culture.** Harvested splenic B-cells from $cMyc^{gfp/gfp}$ mice or C57BL/6 mice 7 days after SRBC immunization were enriched by CD43 negative selection using MACS LS Columns. B-cells from $cMyc^{gfp/gfp}$ mice were cultured with 5 µg/ml anti-CD40 antibody (HM40-3), 12 µg/ml AffiniPure $F(ab')_2$ Fragment Goat Anti-Mouse IgG + IgM (H+L) (Jackson ImmunoResearch) and 2 ng/ml murine IL-4 (PeproTech) in B-cell medium: RPMI 1640 GlutaMAX Supplement medium supplemented with 10% fetal bovine serum, 1% pen-strep solution and 50 µM β-mercaptoethanol. After one hour of short-pulse with the stimulants, these cells were washed in B-cell medium and split into two and cultured with or without the stimulants (anti-CD40 antibody, $F(ab')_2$ and IL-4) for further 8 hours. Cells were surface stained immediately after treatment, and were fixed and permeabilized using a Fixation/Permeabilization Solution Kit.

EdU/BrdU dual labelling and EdU incorporation assays. For EdU/BrdU dual labelling assays, 400 µg EdU (Thermo Fisher Science) in PBS was injected intravenously followed 1 hour later by an intravenous injection of 1 mg BrdU in PBS (BD Biosciences). Spleens were harvested 1, 2.5 and 5 hours after BrdU injection. Cells were surface stained and processed using an APC BrdU Flow Kit (BD Biosciences) first and subsequently with a Click-iT Plus EdU Alexa Fluor 594 Flow Cytometry Assay Kit (Thermo Fisher Science). DNA content was analyzed by staining with 2.5 µM FxCycle Violet Stain (Thermo Fisher Scientific) for 20-30 min in flow cytometry buffer at RT. For EdU incorporation assays, 800 µg EdU in PBS was injected 6 hours before isolation of splenocytes. Cells were surface stained and processed using a Click-iT Plus EdU Pacific Blue Flow Cytometry Assay Kit (Thermo Fisher Science).

Immunofluorescence. 10 µm cryosections were fixed with 4% formaldehyde for 15 min, and permeabilized and blocked with blocking buffer (0.3% Triton X-100, 1% BSA, 5% normal bovine serum in PBS). Spleen

sections were stained with anti-GL7 Alexa 488 (Thermo Fisher Scientific), anti-cMyc Alexa-555 (Abcam), anti-phospho-histone H3 (Ser10) Alexa-647 (D2C8; Cell Signaling Technology) and anti-CD35 BV421 (8C12; BD Biosciences). Slides were analyzed using LSM880 with Airyscan (Zeiss) and processed using ImageJ FIJI (v2.1.0).

scRNAseq and data analysis. cMyc-GFP^{+} GC-B-cells from cMyc$^{gfp/gfp}$ 10 days after NP-CGG in alum immunization were sorted by flow cytometry. Live cells were quantified using the EVE automatic cell counter (NanoEnTek) and normalized between 66,000-333,000 cells/mL. cMyc-GFP^{+} GC-B-cells were from 6 independent cell sortings. The Fluidigm C1 automated microfluidic system was used to capture 96 individual cells onto the 5 – 10 µm C1 IFCs (Fluidigm) for mRNA-seq. The SMARTer Ultra Low Input RNA kit for Sequencing (Takara Bio) was used to reverse transcribe poly(A) RNA and amplify cDNA. ArrayControl RNA Spikes (Thermo Fisher Scientific) were added as recommended in the C1 mRNA protocol. cDNA concentration of each cell sample was quantified using the QuantiFluor dsDNA System (Promega) on Glomax Discover System (Promega) and the quality of random samples was checked on a high-sensitivity DNA chip on a 2100 Bioanalyser System (Agilent). Amplified cDNA at a concentration of 0.10 – 0.3 ng/µL was used for preparing libraries with the Nextera XT DNA Library Preparation Kit (Illumina). The samples were sequenced as paired-end 76bp reads using the HiSeq4000 system to generate typically 4 - 6 million reads. De-multiplexed read sets were assessed with FastQC v0.11.2 and quality and adapter trimmed with Trimmomatic v0.35 (3). After trimming, pairs with either read < 35 bp were discarded. Median pair retention was 91.07%. Surviving pairs were aligned against GRCm38.78 supplemented with ERCC spike sequences with HISAT v2.0.1b (4), duplicates marked with Picard-tools v2.5.0, and reads counted at the gene level with FeatureCounts (subread v1.4.6p1) (5). Expression data were filtered (genes expressed in ≥10% of read sets), normalized using the ERCC spike-in reads (R package scran (6)), and assessed with an in-house pipeline CellStrainer for a variety of metrics: number of reads, percent duplication (as measured by Picard-tools), ratio of spike-in to gene reads, number of features as a factor of number of reads mapped, Spearman's correlation to the mean, number of features lowly expressed (< 10% percentile) as a factor of the number of features detected, number of features highly expressed (≥ 90% percentile) as a factor of the number of features detected, per-feature variance from the mean, proportion of reads mapping

to cytoplasmic genes (GO0005737), and proportion of reads mapping to mitochondrial genes. As previously described, CellStrainer uses these technical metrics to fit a one-class support vector machine (SVM) to the dataset (7). 1000 models were built with a random 10% of the libraries and the entire dataset repeatedly qualified as inlier or outlier for a majority vote decision on whether captures represented valid (live) cells. Separately, original image-calls were standardized to 5 categories: 1 – good capture of single cell, 0 – empty capture, D – single capture of debris, NS – single capture of a non-standard cell, and M – multiple cell capture or co-capture with debris. Further excluding 'M' image-calls, 166 × 1, 3 × D and 36 × NS cells passed CellStrainer. These were used to fit a Michaelis–Menten model of the dataset (R package M3Drop) that, accounting for drop-outs, models whether a feature is variably expressed across the population. Resulting genes were clustered within Qlucore Omics Explorer and groupings interrogated, using the entire gene set, to determine features best describing the population. GSEA was run with Qlucore Omics Explorer.

RNAseq and data analysis. cMyc-GFP^{+} GC-B-cells from cMyc$^{gfp/gfp}$ 7 days after SRBC immunization were sorted by flow cytometry based on the gating shown in Fig. 1D. Total RNA was prepared with TRIzol Reagent (Thermo Fisher Scientific) combined with RNeasy Micro Kit (QIAGEN) and processed using a NEBNext Single Cell/Low Input RNA Library Prep Kit for Illumina (New England BioLabs). RNA-sequencing was carried out on the Illumina HiSeq 4000 platform and typically generated ~30 million 101-bp stranded single-end reads per sample. Adaptor trimming was performed with Trimmomatic/0.36-Java-1.7.0_80 with parameters "LEADING:3 TRAILING:3 SLIDINGWINDOW:4:20 MINLEN:36" (3). The RSEM package (v.1.2.31) (8) in conjunction with the STAR alignment algorithm (v.2.5.2a) (9) was used for the read mapping and gene-level quantification with respect to mouse Ensembl GRCm38 - release 89. The parameters used were: –star-output-genome-bam–forward-prob 0.5. Differential expression analysis was performed with the DESeq2 package (v.1.20.0) (10) within the R programming environment (v.3.5.1). The significance threshold for the identification of differentially expressed genes was set as an adjusted *P* value ≤0.05. 3D PCA was carried out through Qlucore Omics Explorer. GSEA was run with GSEA software v2.2.4 (11). The lists of gene signatures used are: cell cycle; "GO_CELL_CYCLE" (MSigDB), cMyc^{+}AP4^{+} LZ up and AP4^{+} DZ up (12), NFκB activation; "REACTOME_ACTIVATION_OF_NF_KAPPA_IN_B_CELLS" (MSigDB), both MBC up compared to PC and PC up compared to MBC (GSE147098), IRF4 target genes in

PC compared to mature B cell; "SHAFFER_IRF4_TARGETS_IN_PLASMA_CELL_VS_MATURE_B_LYMPHOCYTE" (MSigDB) (13).

Statistics. Statistical parameters including the number of mice, the number of replicates and statistical test used are described in Figure legends. Statistics were calculated using GraphPad Prism software v8. P values ≤ 0.05 were considered statistically significant. Center bars always indicate means. Error bars indicate ±SEM.

Mathematical model. Mathematical modelling has been used to test the consistency of the proposed functional interdependence of the clusters. 5 different sequences of clusters (model 0 to 4) have been tested. Specifically, we used an agent-based model, in which FDCs, B cells and TFHs are distinct objects whose interactions drive the *in silico* GC reaction. *In silico*, $cMyc^+$ is a property attributed to each cell under some conditions, depending on the chosen model (0 to 4). The strategy to include the $cMyc^+$ clusters derived from *in vivo* experiments into the model was linking the property of being in a cluster to an event in the *in silico* GC reaction. With this choice, attribution of $cMyc^+$ to a subset of cells automatically generates the proportions of the clusters. 100 *in silico* simulations for each model were performed to generate the time course of the GC reaction. Differently from *in vivo* experimental data that were obtained from analysis of spleen tissue, the model simulates a single GC reaction. Therefore, experimental data were normalized assigning the maximum value obtained with the simulations (day 6) to the maximum value of the experimental data; all the other data points (days 4, 8, 12) were normalized with respect to this maximum value so that the proportion between the data is maintained. Moreover, the simulation is delayed by two days to match the maximum in the simulation and in the data. This delay is due to the time needed to start the GC reaction after immunization. Each model relies on two parameters and the comparison of the results among the different models and subsequent selection of model 0 was done based on the lowest residual sum of squares (RSS, Eq. 1) and Akaike Information Criterion (AIC, Eq. 2). RSS is an estimator of the difference between the quantitative results of the analysis and the experiments: lower RSS indicates a preferable model.

$$RSS = \sum_{i=1}^{N} \frac{(y_{i_d} - y_{i_s})^2}{{\sigma_{i_d}}^2}, \quad (1)$$

where y_{i_d} and y_{i_s} are the normalized mean at each time point analyzed *in vivo* for the data and the simulation respectively, σ_{i_d} is the standard deviation of the experimental data and *N* is the sample size (*N* = 20, a result of number of *in vivo* samples i.e. 4 - days 4, 6, 8, 12 - times the number of populations to fit i.e. 5 - the total GC population and the four cMyc$^+$ clusters). In the comparison between models, 2 is the minimum distance between the AIC values so that the one with the lowest AIC is actually accepted as an improvement of the other.

$$AIC = N ln(\frac{RSS}{N}) + 2k, \qquad (2)$$

where *k* is the number of parameters used (*k* = 2 for each model).

A differential evolution algorithm was used to tune the parameters and optimize the RSS; the optimized RSS and AIC values for the 5 models are shown in Fig. 2E and SI Appendix, Fig. S2, B - E.

Germinal Centre simulation model. The structure and the parameters used for the Germinal Centre (GC) simulations are explained here. It follows the description of (14) adapted to include novel features introduced since then in (15-17) as well as the description of the different models reproducing the sequence of the cMyc$^+$ clusters. *tc signal*, *selection threshold*, *selected clock*, *individual dif delay* are used below and in the figures to refer to the parameters in the code. For a schematic representation of the cMyc models and related results, see Fig. 2E and SI Appendix, Fig. S2, B - E. Used acronyms are: DZ for dark zone, LZ for light zone, TFH for T follicular helper cell, FDC for follicular dendritic cell.

Space representation. All reactions take place on a three-dimensional discretized space with a rectangular lattice with lattice constant of $\Delta x = 5 \mu m$. Every lattice node can be occupied by a single cell only.

Shape space for antibodies. Antibodies are represented on a four dimensional shape space (18). The shape space is restricted to a size of 10 nodes per dimension, thus, only considering antibodies with a minimum affinity to the antigen. The optimal clone Φ^* is positioned in the center of the shape space. A position on the shape space Φ is attributed to each B cell. Its Hamming distance $\parallel \Phi - \Phi^* \parallel_1$ to the optimal clone is used as a measure for the antigen binding probability. The binding probability is calculated from the Gaussian distribution with width Γ = 2.8 (19):

$$b(\Phi, \Phi^*) = exp(-\frac{\parallel \Phi - \Phi^* \parallel_1^2}{\Gamma^2}). \qquad (1)$$

B cell phenotypes. B cells are distinguished in three different phenotypes: DZ-B-cell, LZ-B-cell and output cells. Cells phenotypes characterize the cell properties: DZ-B-cells can divide, mutate and migrate; LZ-B-cells can migrate and undergo different stages of selection; output cells can only migrate.

Founder cells. The model starts with 250 TFHs, 200 FDCs, 300 stromal cells, and no B-cells. TFHs are randomly distributed on the lattice and occupy a single node each. Stromal cells are restricted to the DZ (see section *Chemokine distribution* for their function). FDCs are restricted to the upper half of the reaction sphere, occupy one node by their soma and have 6 dendrites of 40 μm length each. The presence of dendrites is represented as a lattice-node property and, thus, visible to B-cells. The dendrites are treated as transparent for B-cell or TFH migration such that they do not inhibit cell motility. Each FDC is loaded with 1500 antigen portions distributed onto the lattice-nodes occupied by FDC-soma or FDC-dendrite. One antigen portion corresponds to the number of antigen molecules taken up by a B-cell upon successful contact with an FDC.

B-cell influx rate. Seeder B-cells enter the model in the first three days of the reaction (i.e. 2-5 days post-immunization) with a rate of 3 cells/hour. Thus, 216 B-cells seed the GC and generate the founder cell population starting the first round of affinity maturation. In this expansion phase B-cells divide 7 times.

DZ-B-cell division. The average cell cycle duration of 7 hours of DZ-B-cells is varied for each B cell according to a Gaussian distribution. This is needed to get desynchronization of B-cell division. The cell cycle is decomposed into four phases (G_1, S, G_2, M) in order to localize mitotic events if this is needed. Each founder B cell divides a number of times before differentiating to the LZ phenotype for the first time. Six divisions was the number of divisions found in response to the extreme stimulus with anti-DEC205-OVA (14, 20). Each selected B-cell divides a number of times determined by the interaction with TFHs (see below, LZ-B-cell selection). The parameters of the interaction with TFHs are tuned such that the mean number of divisions is in the range of two (21). This value is required in order to maintain a DZ to LZ ratio in the range of two (14, 20). A division requires free space on one of the Moore neighbors of the dividing cells. Otherwise the division is postponed until a free Moore neighbor is available. At every division the encoded antibody can mutate with a probability of 0.5 (22). This corresponds to a shift in the shape space to a von Neumann neighbor in a random direction. Upon selection by TFH the mutation probability is individually reduced from $m_{max} = 0.5$ down to $m_{min} = 0$ in an affinity-dependent way following Eq. 2

$$m(b) = m_{max} - (m_{max} - m_{min})b, \qquad (2)$$

with b from Eq. 1 (23). Thus, after recycling DZ-B-cells can acquire reduced mutation probabilities. This mechanism is motivated by the observation that BCR internalization enhances the activation of the kinase Akt (24) which, in turn, suppresses activation induced cytosine deaminase (AID) (25). AID is required for somatic hypermutation, such that this provides an affinity-dependent down-regulation of the mutation frequency (26). However, there is no formal proof of this mechanism.

LZ-B-cell selection. The different states for LZ-B-cells are: *unselected*, *FDC-contact*, *FDC-selected*, *Tfh-contact*, *Tfh-selected*, *apoptotic*.

Unselected. LZ-B-cells freshly differentiated from DZ-B-cells get unselected state, migrate and search for contact with FDCs for 0.7 hours in order to collect antigen. If an FDC soma or dendrite is present at the position of the B-cell, the B-cell attempts to establish contact to the FDCs with probability *b* in Eq. 1. If the available number of antigen portions at the specific FDC site drops below 20 the binding probability *b* is linearly reduced with the number of available portions. If B-cell succeeds, its state switches to FDC-contact; otherwise the B-cell continues to migrate. Further binding-attempts are prohibited for 1.2 minutes. At the end of the antigen collection period, B-cells switch to the state FDC-selected. If a LZ-B-cell fails to collect any antigen at this time it switches to the state apoptotic.

FDC-contact. LZ-B-cells remain immobile (bound) for 3 minutes (27) and then return to the state unselected. The counter for the number of successful antigen uptake events is increased by one and the FDC reduces its locally available antigen portions by one.

FDC-selected. B-cells search for contact with TFH. If they meet a TFH they switch to the state TFH-contact. FDC-selected B-cells coming from TFH-contact state, switch to TFH-selected state if TFH-signaling time is above or equal to 18 minutes (*collected signal* ≥ *selection threshold* = 0.3 h), otherwise B-cells state switch to apoptotic state.

Tfh-contact. Multiple short T-B interactions (17, 28) are used in the simulation. During each interaction, LZ-B cells remain immobile for 6 minutes. In this time the bound TFH, which may also be bound to other B-cells, polarizes to the bound B-cell with highest number of successful antigen uptakes. After the binding time, B-cells state is set back to FDC-selected. B-cells try to accumulate TFH signals for 3 hours.

Tfh-Selected. LZ-B-cells keep the LZ phenotype for six hours on average (*individual dif delay*) and desensitize for CXCL13. During that time they re-enter cell cycle and progress through the cell cycle phases.

Then, 10% of selected B-cells differentiate to output cells and leave the GC through the light zone and 90% of the selected B-cells recycle back to the DZ phenotype with a rate of 1/6 minutes (BASE model (14)). The latter B-cells memorize the amount of collected antigen as well as the cell cycle phase they have achieved by this time. The number of divisions *P(A)* the recycled B-cells will do is derived from the amount of collected antigen A, which reflects the amount of pMHC presented to TFH and the affinity of the BCR for the antigen, as follows:

$$P(A) = P_{min} + (P_{max} - P_{min})\frac{A^{n_P}}{A^{n_P}+K_P^{n_P}}. \quad (3)$$

The more antigen was collected by the B-cell, the more divisions are induced. We set the minimum number of divisions to one ($P_{min} = 1$) in order to avoid recycling events without further division. It is limited by six divisions in the best case ($P_{max} = 6$) which is motivated by anti-DEC205-OVA experiments in which DEC205$^{+/+}$ B cells received abundant antigen which increased pMHC presentation to a maximum (20). The population dynamics *in vivo* and *in silico* only matched when the number of divisions was increased to six in the simulation (14) suggesting that the strongest possible pMHC presentation to TFH induces six divisions. The Hill-coefficient was set to $n_P = 2$. The half value K_P remained to be determined, which denotes the amount of antigen collected by B-cells at which the number of divisions becomes half maximal. The number of collected antigen portions varies between zero and a maximum determined by the duration of the antigen collection phase, the duration of each B-cell interaction with FDCs, and the migration time between two antigen presenting sites. The numbers of successful B cell-FDC encounters as observed in the simulations served as estimate of A_{max}. Low affinity B-cells had zero or one antigen uptake event, while high-affinity cells took up between 5 and 10 portions. For an intermediate antigen uptake of $A_0 = 4.5$, the resulting number of divisions has to be $P_0 = 2$ in order to be in agreement with the mean number of divisions in the range of two (21), which leads to the condition in Eq. 4

$$K_P \approx A_0(\frac{P_{max}-P_{min}}{P_0-P_{min}} - 1)^{1/n_P} = 9. \quad (4)$$

Apoptotic. LZ-B-cells remain on the lattice for 6 hours before they are deleted. They continue to be sensitive to CXCL13 during this time.

Chemokine distribution. Two chemokines CXCL12 and CXCL13 are considered. CXCL13 is produced by FDCs in the LZ with 10nMol per hour and FDC while CXCL12 is produced by stromal cells in the DZ with

400nMol per hour and stromal cells. As both cell types are assumed to be immobile, chemokine distributions were pre-calculated once and the resulting steady state distributions were used in all simulations.

Chemotaxis. DZ- and LZ-B-cells regulate their sensitivity to CXCL13 and CXCL12, respectively. This is true in all B-cell states unless stated otherwise. All B-cells move with a target speed of $7.5 \mu m/min$. This leads to a slightly lower observable average speed of $\approx 6 \mu m/min$. B-cells have a polarity vector that determines their preferential direction of migration. The polarity vector $\vec{p}$ is reset every 1.5 minutes into a new direction using the chemokine distribution c as

$$\vec{p} = \vec{p}_{rand} + \frac{\alpha}{1+exp\{\kappa(K_{1/2} - \Delta x|\vec{\nabla} c|)\}} \frac{\vec{\nabla} c}{|\vec{\nabla} c|}, \quad (5)$$

where $\vec{p}_{rand}$ is a random polarity vector and the turning angle is sampled from the measured turning angle distribution (29). $\alpha = 10$ determines the relative weight of the chemotaxis and random walk, $K_{1/2} = 2 \cdot 10^{11} Mol$ determines the gradient of half maximum chemotaxis weight, and $\kappa = 10^{10}/Mol$ determines the steepness of the weight increase. B-cells de- and re-sensitize for their respective chemokine depending on the local chemokine concentration: The desensitization threshold is set to 6nMol and 0.08nMol for CXCL12 and CXCL13, respectively, which avoids cell clustering in the center of the zones. The resensitization threshold is set at 2/3 and 3/4 of the desensitization threshold for CXCL12 and CXCL13, respectively. B-cells can only migrate if the target node is free. If occupied and the neighbor cell is to migrate in the opposite direction (negative scalar product of the polarity vectors) both cells are exchanged with a probability of 0.5. This exchange algorithm avoids lattice artifacts leading to cell clusters. TFH do random walk with a preferential directionality to the LZ: The polarity vector $\vec{p}$ of TFH is determined from a mixture of random walk $\vec{r}$ and the direction of the LZ $\vec{n}$ by

$$\vec{p} = (1 - \alpha')\vec{r} + \alpha'\vec{n}, \quad (6)$$

where $\alpha' = 0.1$ is the weight of chemotaxis. This weight leads to a dominance of random walk with a tendency to accumulate in the LZ as found in experiment. T-cells migrate with an average speed of and repolarize every 1.7 minutes. Output cell motility is derived from plasma$10 \mu m/min$ cell motility data to be $3 \mu m/min$ (29) with a persistence time of 0.75 minutes.

cMyc-models. Schematic representation in Fig. 2E and SI Appendix, Fig. S2, B-E of results section and the text. The clusters are enumerated and cited below in the form (#*X*), where *X* is the number of the cluster.

- Model 0: B cells that passed the threshold of the TFH signal to be TFH-selected (*collected signal* ≥ 0.5) switch to $cMyc^{+}$early (cluster #3). For $cMyc^{+}$early cells, the decision of differentiating into output cells or recycling to DZ-B-cells is taken at the time of selection (*TC-selection*). Cluster #3 B-cells switch to $cMyc^{+}$prePB (cluster #1) if they differentiate to output cells and to $cMyc^{+}$ late (cluster #4) if they recycle as DZ-B-cells. At the moment cells in cluster #4 differentiate to CB they switch to $cMyc^{+}$DZ (cluster #2) and the latter lose cMyc expression after some time. The two free parameters used in model 0 to fit the data are the threshold for the collected TFH signal above which B-cells are attributed to the cluster #3 and the time spent in the cluster #2.
- Model 1: TFH-selected B-cells switch to $cMyc^{+}$prePB (cluster #1) if they differentiate to output cells or $cMyc^{+}$early (cluster #3) if they recycle as DZ-B-cells. With a *delay*, cells in cluster #3 switch to $cMyc^{+}$late (cluster #4). Cells in cluster #4 switch in $cMyc^{+}$DZ (cluster #2) when they differentiate to CB and the latter lose cMyc expression after some time. The two free parameters used in model 1 to fit the data are the time B-cells spend in the cluster #3, before switching to the cluster-#4 and the time spent in the cluster #2.
- Model 2: Analogous setup to model 0, with interchanged role between cluster #3 and #4. B-cells that passed the threshold of the TFH signal to be TFH-selected (*collected signal* ≥ 0.5) switch to $cMyc^{+}$late (cluster #4). Cluster #4 B-cells switch to $cMyc^{+}$prePB (cluster #1) if they differentiate to output cells and to $cMyc^{+}$early (cluster #3) if they recycle as DZ-B-cells. When cells in cluster #3 differentiate to CB or when they reach the DZ, whatever happens first, they switch in $cMyc^{+}$DZ (cluster #2) and the latter lose cMyc expression after some time. The two free parameters used in model 2 to fit the data are the threshold for the collected TFH signal above which B-cells are attributed to the cluster #4 and the time spent in the cluster #2.
- Model 3: Analogous setup to model 1, with interchanged role between cluster #3 and #4. TFH-selected B-cells switch to $cMyc^{+}$prePB (cluster #1) if they differentiate to output cells or $cMyc^{+}$late (cluster #4) if they recycle as DZ-B-cells. With a *delay*, cells in cluster #4 switch to $cMyc^{+}$early (cluster #3). Cells in cluster #3 switch in $cMyc^{+}$DZ (cluster #2) when they differentiate to CB and the latter lose cMyc expression after

some time. The two free parameters used in model 3 to fit the data are the time B-cells spend in the cluster #4, before switching to the cluster #3 and the time spent in the cluster #2.

- Model 4: TFH-selected B-cells subdivide in two populations. Recycling cells (90%) form the cluster of cMyc^{+}late (cluster #4), the rest is further subdivided depending on a percentage in cMyc^{+}prePB cluster (cluster #1) and cMyc^{+}early (cluster #3). Cells in cluster #1 lose cMyc expression at the time of differentiation while cells in cluster #3 become cMyc^{neg} when they reach the DZ. Cells in cluster #4 switch in cMyc^{+}DZ (cluster #2) when they differentiate to CB and the latter lose cMyc expression after some time. The two free parameters used in model 4 to fit the data are the percentage of B-cells attributed to the cluster #1 and the time spent in the cluster #2.

In all cMyc models, apoptotic cells lose cMyc expression.

Fig S1. Detailed analysis of scRNAseq and cell numbers of cMyc⁺ GC-B-cell subpopulations.

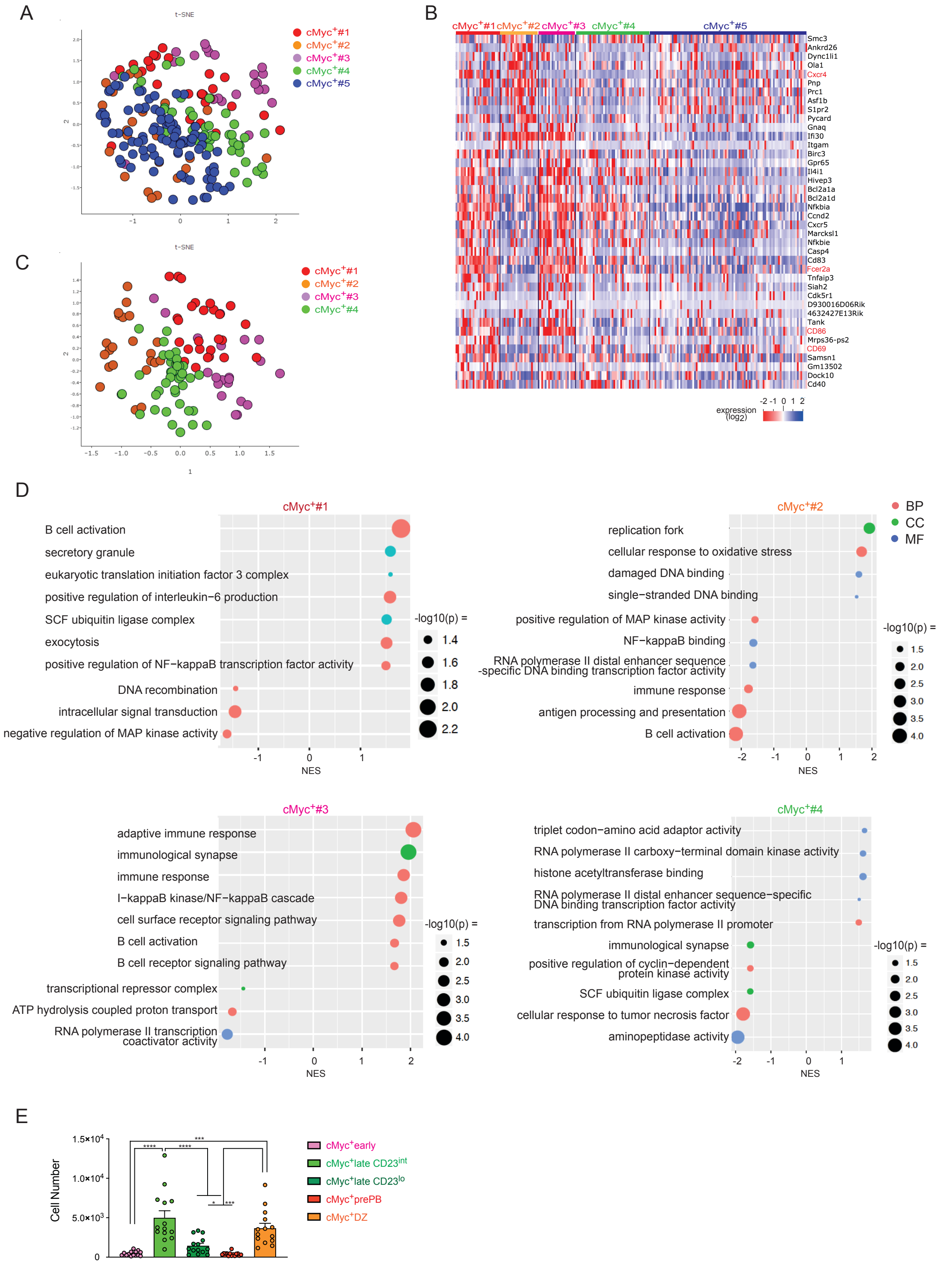

Figure S1. Detailed analysis of scRNAseq and cell numbers of cMyc^{+} GC-B-cell subpopulations.

(A) t-SNE plot showing the five identified cMyc^{+} GC-B-cell clusters of 205 individual cell transcriptomes. Each dot represents a single cell colored by cluster assignment. **(B)** Heat map showing unsupervised clustering with DEGs from scRNAseq between five cMyc^{+} GC-B-cell clusters by multi-group comparison ($p \leq 0.0005$). **(C)** t-SNE plot showing the four cMyc^{+} GC-B-cell clusters of 113 individual transcriptomes. Each point is a single cell colored by cluster assignment. **(D)** Dot plots showing representative results of GSEA. Only pathways that are enriched at threshold of $p \leq 0.05$ and NES > 1.44 are shown. NES; normalized enrichment score, BP (pink); biological process, CC (green); cellular component, MF (blue); molecular function. **(E)** Enumeration of the five cMyc^{+} GC-B-cell subpopulations. Splenic GC-B-cell response to SRBC on day 7 in cMyc$^{gfp/gfp}$ mice. Data are derived from more than three independent experiments with 14 mice. Error bars indicate SEM. Statistics were calculated with one-way ANOVA. * $p < 0.05$, *** $p < 0.001$, **** $p < 0.0001$.

Figure S2. Enriched pathways and *in silico* models for temporal dynamics of cMyc+ GC-B-cell subpopulations.

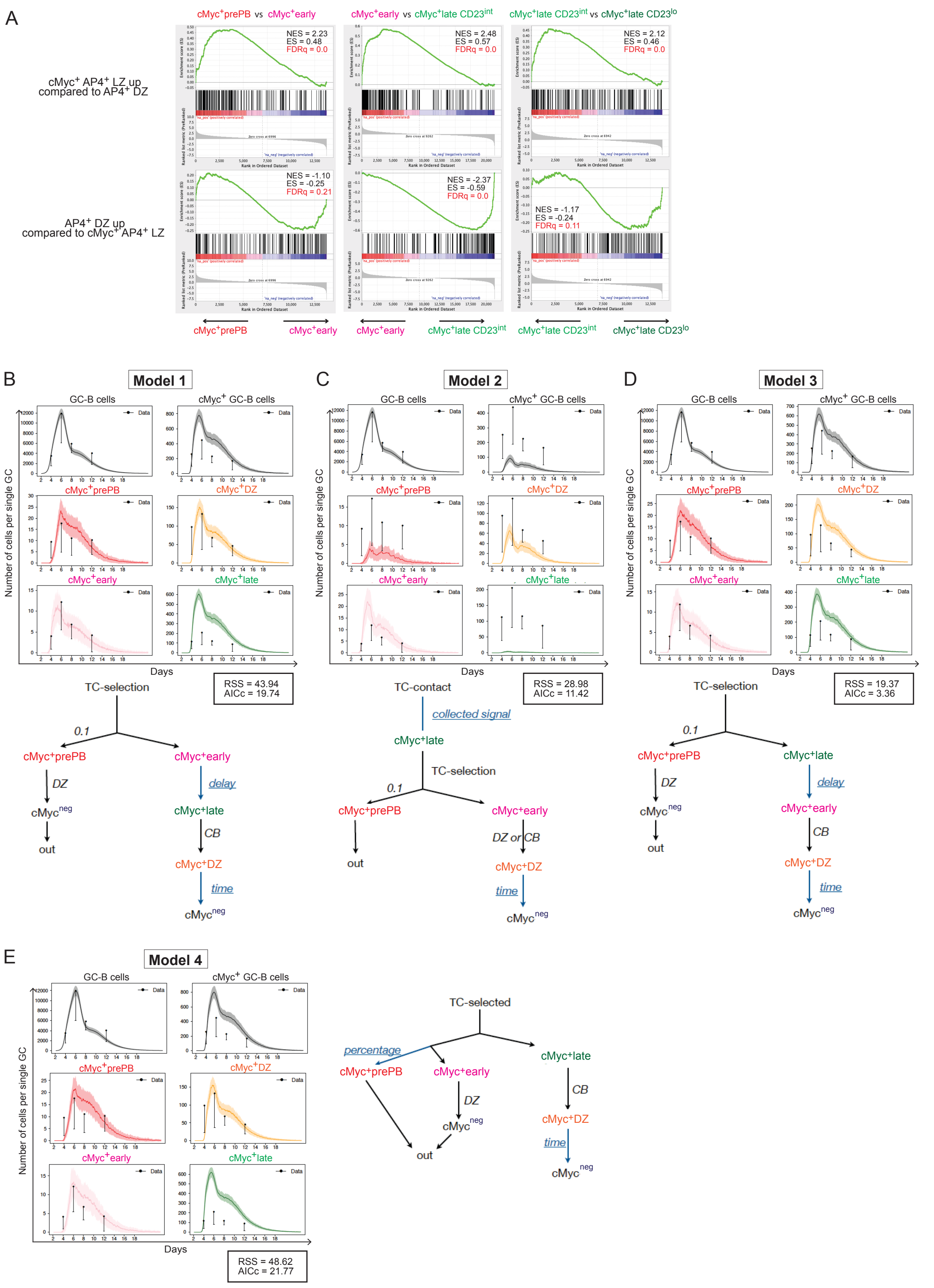

Figure S2. Enriched pathways and *in silico* models for temporal dynamics of cMyc$^+$ GC-B-cell subpopulations.

(A) GSEA of differential gene expression in a cMyc$^+$ GC-B-cell subpopulation vs. the other (described in the Figure) for gene sets: "cMyc$^+$AP4$^+$ LZ upregulated compared to AP4$^+$ DZ" and "AP4$^+$ DZ upregulated compared to cMyc$^+$AP4$^+$ LZ". Statistical significance (FDRq < 0.25) is highlighted in red. NES; normalized enrichment score, ES; enrichment score, FDR; false discovery rate. *In silico* kinetics were obtained from simulations based on model 1 **(B)**, model 2 **(C)**, model 3 **(D)** and model 4 **(E)** and were implemented to reproduce the dynamics of cMyc$^+$ GC-B-cell subpopulations determined by *in vivo* experiments. The kinetics of the GC response *in silico* are plotted over the *in vivo* results for cell number of GC-B-cells, cMyc$^+$ GC-B-cells and cMyc$^+$ GC-B-cell subpopulations. Residual sum of squares (RSS), Akaike information criterion (AICc) values and working model of the cMyc$^+$ GC-B-cell subpopulation dynamics *in silico* are shown at bottom (B - E). Free parameters used to fit the model are shown in blue letters. All the data points were normalized with respect to the maximum value obtained in the simulation of the GC-B-cell kinetics in GC-B-cell numbers shown at the top left plot. Mean (full lines) and standard deviation (shaded area) of 100 simulations are shown. Black dots and colored dots represent *in vivo* data.

Fig. S3. Cell cycle progression of cells in cMyc⁺ GC-B-cell subpopulations.

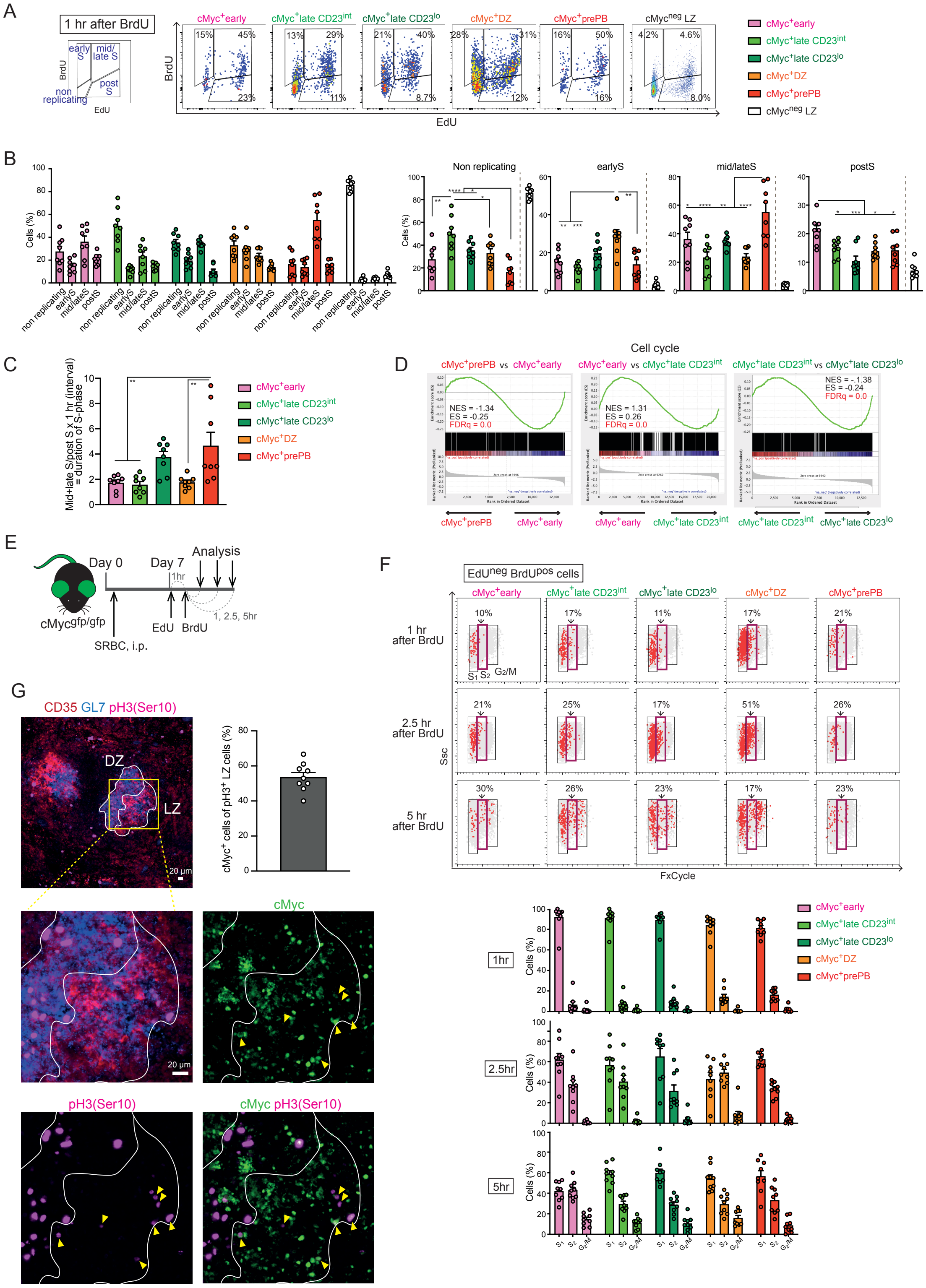

Figure S3. Cell cycle progression of cells in cMyc$^+$ GC-B-cell subpopulations.

(A) Representative flow cytometric plots of EdU incorporation vs. BrdU incorporation in the cMyc$^+$ GC-B-cell subpopulations and cMyc^{neg} LZ-B-cell population. **(B)** Percent of cells of the cMyc$^+$ GC-B-cell subpopulations and cMyc^{neg} LZ population in the indicated phases of the cell cycle. **(C)** Mid+late S/post S ratio in the cMyc$^+$ GC-B-cell subpopulations. **(D)** GSEA of differential gene expression in a cMyc$^+$ GC-B-cell subpopulation vs. the other for gene sets: "Cell cycle". Statistical significance (FDRq < 0.25) is highlighted in red. NES; normalized enrichment score, ES; enrichment score, FDR; false discovery rate. **(E)** Experimental design for (F). **(F)** Representative flow cytometric plots of FxCycle vs. Ssc in EdU^{neg}BrdU$^+$ cells that have initiated S-phase of the cMyc$^+$ GC-B-cell subpopulations after 1, 2.5 and 5 hr BrdU incorporation (top). Percent of cells in the indicated phases of the cell cycle of the cMyc$^+$ subpopulations after 1, 2.5 and 5 hr BrdU incorporation (bottom). **(G)** Representative images of GCs in spleens from C57/BL6 mice at day 7 after SRBC immunization. Data are representative of imaged GCs from 3 mice. Percentages of cMyc$^+$ phospho-histone H3 (pH3)$^+$ LZ-B cells of total pH3$^+$ LZ cells (top right). Each dot represents one GC. Splenic GC-B-cell response to SRBC on day 7 in cMyc$^{gfp/gfp}$ mice (A-C and F). Data are from three independent experiments with 8 mice (B and C) or 9 mice (F) at each time point. Error bars indicate SEM. Statistics were calculated with one-way ANOVA. * $p < 0.05$, ** $p < 0.01$, *** $p < 0.001$, **** $p < 0.0001$.

Fig. S4. Levels of cMyc expression and apoptosis in cMyc+ GC-B-cell subpopulations of SRBC-immunized mice.

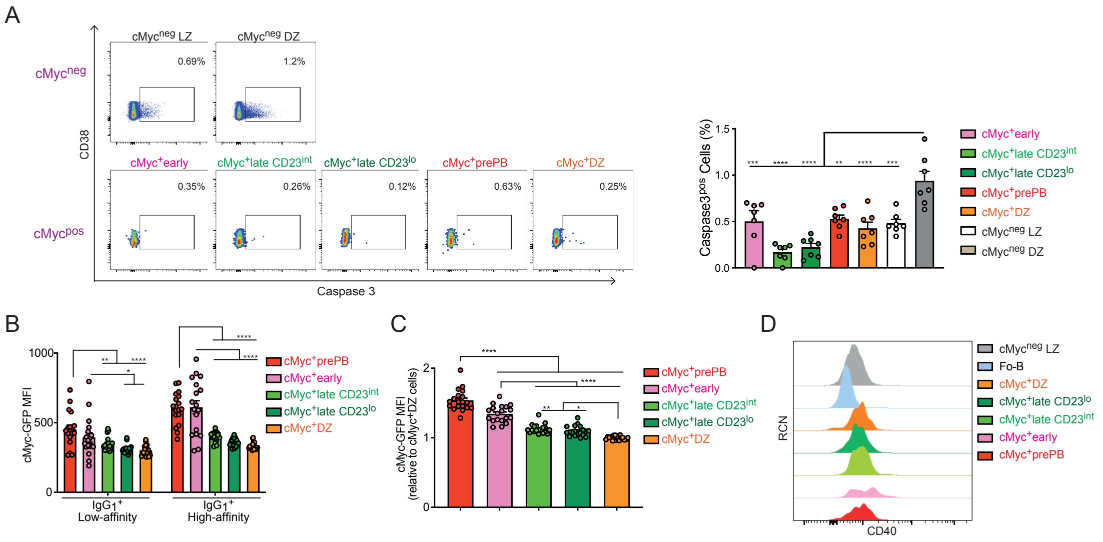

Figure S4. Levels of cMyc expression and apoptosis in $cMyc^+$ GC-B-cell subpopulations of SRBC-immunized mice.

(A) Representative flow cytometric plots of active caspase 3 vs. CD38 in the indicated GC-B-cell populations (left). Percent of caspase 3^{pos} cells in the indicated cell populations (right). Splenic GC-B-cell response to SRBC on day 7 in $cMyc^{gfp/gfp}$ mice. **(B)** cMyc-GFP MFI values of the $cMyc^+$ GC-B-cell subpopulations. SW_{HEL} $cMyc^{gfp/gfp}$ donor derived ($CD45.1^{neg}CD45.2^+$) HEL-specific cells 8 days after $HEL^{3\times}$-SRBC immunization were divided into IgG_1^{pos} high-affinity and low-affinity. Statistics calculated with one-way ANOVA between $cMyc^+$ GC-B-cell subpopulations within the low and high-affinity cells. **(C)** cMyc-GFP MFI values of the five $cMyc^+$ GC-B-cell subpopulations (relative to $cMyc^+DZ$ values). **(D)** Representative flow cytometric histograms of CD40 vs. RCN. Splenic GC-B-cell response to SRBC on day 7 in $cMyc^{gfp/gfp}$ mice (A - C). Data are derived from two independent experiments with 7 mice (A), 18 mice (B) or 19 mice (C). Error bars indicate SEM. Statistics were calculated with one-way ANOVA. * $p < 0.05$, ** $p < 0.01$, *** $p < 0.001$, **** $p < 0.0001$.

Fig. S5. Blimp1/IRF4 expression in cMyc⁺ GC-B-cell subpopulations and the proportion of MBC precursors over time.

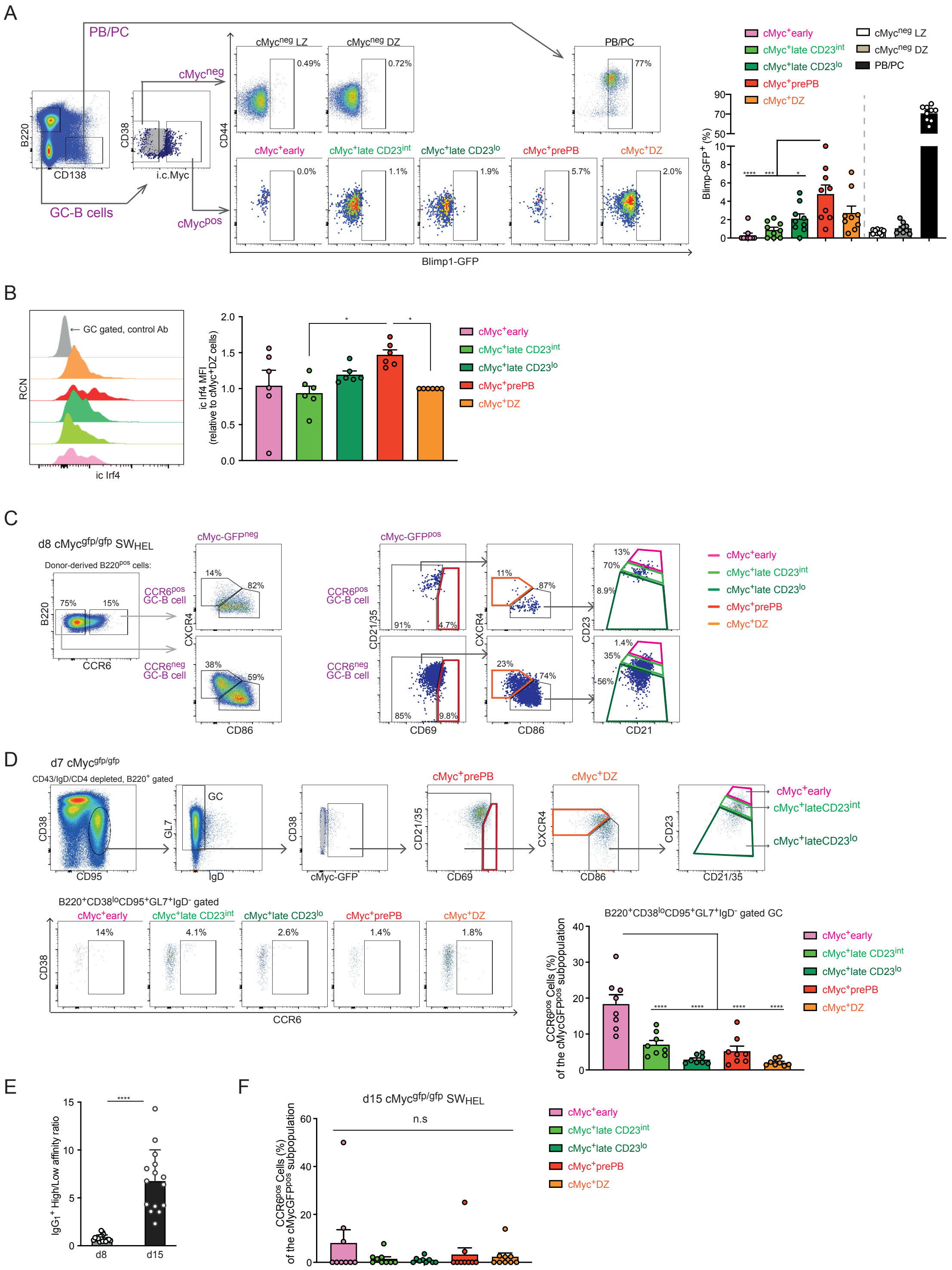

Figure S5. Blimp1/IRF4 expression in cMyc$^+$ GC-B-cell subpopulations and the proportion of MBC precursors over time.

The data are from SW_{HEL} $cMyc^{gfp/gfp}$ donor derived GC-B-cells after $HEL^{3\times}$-SRBC immunization (C, E and F) or splenic GC-B-cells of the $cMyc^{gfp/gfp}$ mice after SRBC immunization (B and D). **(A)** Representative flow cytometric plots of Blimp1-GFP expression in splenic GC-B cells derived from $prdm1^{wt/gfp}$ mice 7 days after SRBC immunization (left). Percentages of cells in the Blimp1-GFP$^+$ gate (right). **(B)** Representative flow cytometric histograms of Irf4 vs. RCN (left). Relative Irf4 MFI values of the cMyc$^+$ GC-B-cell subpopulations (right). **(C)** B-cells were divided based on CCR6 and GC marker expression. GC-B-cells were further divided based on cMyc expression, followed by delineation of the five cMyc$^+$ GC-B-cell subpopulations. Representative flow cytometric plots are shown. **(D)** Representative flow cytometry plots illustrating the gating strategy to delineate the five cMyc$^+$ GC-B-cell subpopulations with two more markers (GL7 and IgD) than the usual gating strategy shown in Fig. 1D. Splenic GC-B-cells of C57BL/6 mice are shown in grey dots as cMyc-GFP^{neg} control cells (top). Representative flow cytometric plots of CCR6 expression in the cMyc$^+$ GC-B-cell subpopulations under this GC gating strategy. Percentage of CCR6^{pos} cells in the cMyc$^+$ GC-B-cell subpopulations on day 7. **(E)** IgG_1^{pos} high/low $HEL^{3\times}$ binding ratio in GC-B cells on day 8 and 15. **(F)** Percentage of CCR6^{pos} cells in the cMyc$^+$ GC-B-cell subpopulations on day 15. Data are from two independent experiments with 6 mice (B), 8 mice (D) or 9 mice (F), more than three independent experiments with 9 mice (A), 17 mice (E, d8) or 15 mice (E, d15). Error bars indicate SEM. Statistics were calculated with unpaired student's t-test (E), unless otherwise with one-way ANOVA (A - C and E). * $p < 0.05$, *** $p < 0.001$, **** $p < 0.0001$. n.s = not significant.

Fig. S6. Working model for GC positive selection

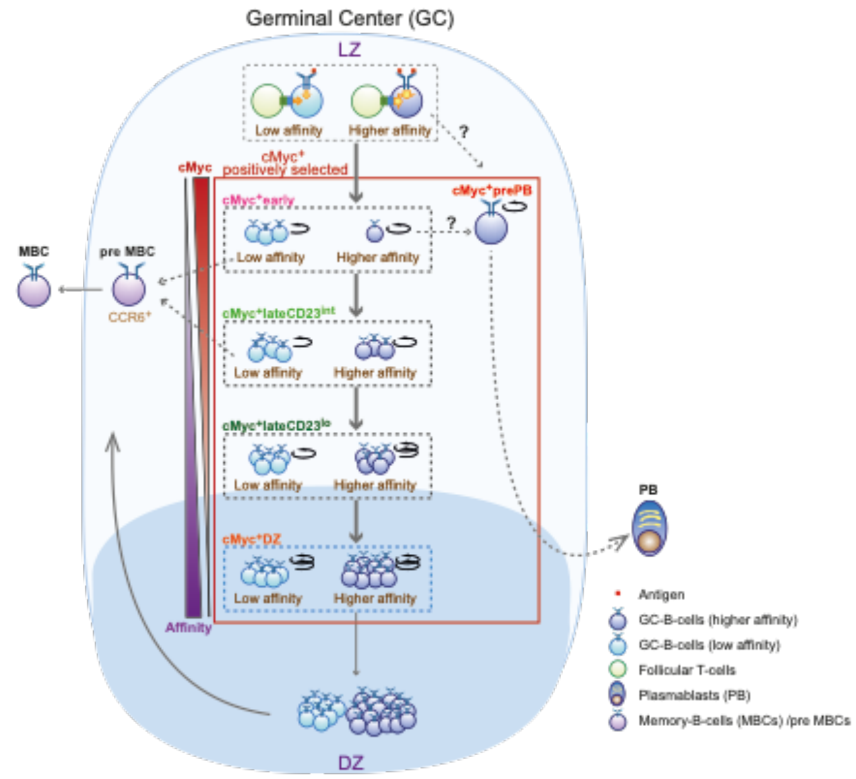

Figure S6: Working model for GC positive selection.

GC positive selection starts permissively to retain a large proportion of low-affinity clones that are protected from apoptosis despite their low-affinity BCR. Low-affinity enriched less-proliferative $CCR6^+$ cells within the $cMyc^+$early and $cMyc^+$late$CD23^{int}$ LZ-B-cell subpopulations differentiate into MBCs. $cMyc^+$ LZ-B-cells change their phenotypes concomitantly with reduction of CD23 expression while BCR affinity within the $cMyc^+$ LZ-B-cell compartment is enhanced by affinity-dependent proliferation. A high-affinity clone enriched subpopulation differentiates into PBs shortly after they are positively selected.

SI References

1. D. Paus *et al.*, Antigen recognition strength regulates the choice between extrafollicular plasma cell and germinal center B cell differentiation. *J Exp Med* **203**, 1081-1091 (2006).
2. A. Kallies *et al.*, Plasma cell ontogeny defined by quantitative changes in blimp-1 expression. *J Exp Med* **200**, 967-977 (2004).
3. A. M. Bolger, M. Lohse, B. Usadel, Trimmomatic: a flexible trimmer for Illumina sequence data. *Bioinformatics* **30**, 2114-2120 (2014).
4. D. Kim, B. Langmead, S. L. Salzberg, HISAT: a fast spliced aligner with low memory requirements. *Nat Methods* **12**, 357-360 (2015).
5. Y. Liao, G. K. Smyth, W. Shi, featureCounts: an efficient general purpose program for assigning sequence reads to genomic features. *Bioinformatics* **30**, 923-930 (2014).
6. A. T. Lun, D. J. McCarthy, J. C. Marioni, A step-by-step workflow for low-level analysis of single-cell RNA-seq data with Bioconductor. *F1000Res* **5**, 2122 (2016).
7. T. Donnarumma *et al.*, Opposing Development of Cytotoxic and Follicular Helper CD4 T Cells Controlled by the TCF-1-Bcl6 Nexus. *Cell Rep* **17**, 1571-1583 (2016).
8. B. Li, C. N. Dewey, RSEM: accurate transcript quantification from RNA-Seq data with or without a reference genome. *BMC Bioinformatics* **12**, 323 (2011).
9. A. Dobin *et al.*, STAR: ultrafast universal RNA-seq aligner. *Bioinformatics* **29**, 15-21 (2013).
10. M. I. Love, W. Huber, S. Anders, Moderated estimation of fold change and dispersion for RNA-seq data with DESeq2. *Genome Biol* **15**, 550 (2014).
11. A. Subramanian *et al.*, Gene set enrichment analysis: a knowledge-based approach for interpreting genome-wide expression profiles. *Proc Natl Acad Sci U S A* **102**, 15545-15550 (2005).
12. C. Chou *et al.*, The Transcription Factor AP4 Mediates Resolution of Chronic Viral Infection through Amplification of Germinal Center B Cell Responses. *Immunity* **45**, 570-582 (2016).
13. A. L. Shaffer *et al.*, IRF4 addiction in multiple myeloma. *Nature* **454**, 226-231 (2008).
14. M. Meyer-Hermann *et al.*, A theory of germinal center B cell selection, division, and exit. *Cell Rep* **2**, 162-174 (2012).
15. S. C. Binder, M. Meyer-Hermann, Implications of Intravital Imaging of Murine Germinal Centers on the Control of B Cell Selection and Division. *Front Immunol* **7**, 593 (2016).
16. M. Meyer-Hermann, Overcoming the dichotomy of quantity and quality in antibody responses. *J Immunol* **193**, 5414-5419 (2014).
17. I. Papa *et al.*, TFH-derived dopamine accelerates productive synapses in germinal centres. *Nature* **547**, 318-323 (2017).
18. A. S. Perelson, G. F. Oster, Theoretical studies of clonal selection: minimal antibody repertoire size and reliability of self-non-self discrimination. *J Theor Biol* **81**, 645-670 (1979).
19. M. Meyer-Hermann, A. Deutsch, M. Or-Guil, Recycling probability and dynamical properties of germinal center reactions. *J Theor Biol* **210**, 265-285 (2001).
20. G. D. Victora *et al.*, Germinal center dynamics revealed by multiphoton microscopy with a photoactivatable fluorescent reporter. *Cell* **143**, 592-605 (2010).
21. A. D. Gitlin, Z. Shulman, M. C. Nussenzweig, Clonal selection in the germinal centre by regulated proliferation and hypermutation. *Nature* **509**, 637-640 (2014).
22. C. Berek, A. Berger, M. Apel, Maturation of the immune response in germinal centers. *Cell* **67**, 1121-1129 (1991).
23. K. M. Toellner *et al.*, Low-level hypermutation in T cell-independent germinal centers compared with high mutation rates associated with T cell-dependent germinal centers. *J Exp Med* **195**, 383-389 (2002).
24. A. Chaturvedi, R. Martz, D. Dorward, M. Waisberg, S. K. Pierce, Endocytosed BCRs sequentially regulate MAPK and Akt signaling pathways from intracellular compartments. *Nat Immunol* **12**, 1119-1126 (2011).
25. S. A. Omori *et al.*, Regulation of class-switch recombination and plasma cell differentiation by phosphatidylinositol 3-kinase signaling. *Immunity* **25**, 545-557 (2006).
26. M. L. Dustin, M. Meyer-Hermann, Immunology. Antigen feast or famine. *Science* **335**, 408-409 (2012).
27. T. A. Schwickert *et al.*, In vivo imaging of germinal centres reveals a dynamic open structure. *Nature* **446**, 83-87 (2007).

28. Y. Wang *et al.*, Germinal-center development of memory B cells driven by IL-9 from follicular helper T cells. *Nat Immunol* **18**, 921-930 (2017).
29. C. D. Allen, T. Okada, H. L. Tang, J. G. Cyster, Imaging of germinal center selection events during affinity maturation. *Science* **315**, 528-531 (2007).
